# Greater morphological and primary metabolic adaptations in roots contribute to phosphate-deficiency tolerance in the bread wheat cultivar Kenong199

**DOI:** 10.1186/s12870-021-03164-6

**Published:** 2021-08-19

**Authors:** Lu Zheng, Mohammad Rezaul Karim, Yin-Gang Hu, Renfang Shen, Ping Lan

**Affiliations:** 1 State Key Laboratory of Soil and Sustainable Agriculture, Institute of Soil Science, Chinese Academy of Sciences, Nanjing, 210008 China; 2 University of Chinese Academy of Sciences, Beijing, 100049 China; 3 State Key Laboratory of Crop Stress Biology for Arid Areas, College of Agronomy, Northwest A&F University, Yangling, 712100 China

**Keywords:** Phosphate deficiency, Wheat, Roots, Signaling, Metabolites

## Abstract

**Background:**

Phosphate (Pi) deficiency severely affects crop growth and productivity, including wheat, therefore it is necessary to develop cultivars with enhanced Pi-deficiency tolerance. However, the underlying mechanism of Pi-deficiency tolerance in wheat is still elusive. Two contrasting wheat cultivars, low-Pi tolerant Kenong199 (KN199) and low-Pi sensitive Chinese Spring (CS) were used to reveal adaptations in response to Pi deficiency at the morphological, physiological, metabolic, and molecular levels.

**Results:**

KN199 was more tolerant to Pi deficiency than CS with significantly increased root biomass and R/S ratio. Root traits, the total root length, total root surface area, and total root volume, were remarkably enhanced by Pi deficiency in KN199. The shoot total P and soluble Pi concentrations of KN199 were significantly higher than those of CS, but not in roots. In KN199, high Pi level in shoots is a higher priority than that in roots under Pi deficiency. It was probably due to differentially regulation in the miR399-mediated signaling network between the shoots of the two cultivars. The Pi deficiency-induced root architecture adaptation in KN199 was attributed to the regulation of the hormone-mediated signaling (ethylene, gibberellin, and jasmonates). The expression of genes associated with root development and Pi uptake was enhanced in KN199. Some primary metabolites (amino acids and organic acids) were significantly accumulated in roots of KN199 under Pi deficiency.

**Conclusions:**

The low-Pi tolerant wheat cultivar KN199 possessed greater morphological and primary metabolic adaptations in roots than CS under Pi deficiency. The adaption and the underlying molecular mechanisms in wheat provide a better understanding of the Pi-deficiency tolerance and the strategies for improving Pi efficiency in wheat.

**Supplementary Information:**

The online version contains supplementary material available at 10.1186/s12870-021-03164-6.

## Background

Phosphorus (P) is an essential macronutrient required for plant growth and development. It is one of the main constituents of nucleic acids, phospholipids, ATP and phosphorylated enzymes, and so on [1]. Phosphorus in plants is acquired from the soil mainly by uptake of inorganic phosphate (Pi). However, P exists as various insoluble phosphate minerals or organic complexes and unevenly distributed in soils [2]. Compared with other macronutrients, P is the most immobile, inaccessible, and unavailable one. Low Pi availability is often a primary limiting factor for plant growth, development, productivity, and grain quality in agricultural systems [3].

To adapt to low-Pi environments, plants have evolved several complex and elaborate responsive and adaptive mechanisms to maintain Pi homeostasis, including root system architecture (RSA) modification, organic acid exudation, production and secretion of phosphatases, and association of the roots with soil microbes, and so on [4, 5]. Meanwhile, plants modulate primary, secondary, and lipid metabolism for high Pi uptake and utilization efficiency. Among them, RSA is a crucial component for enhanced root exploration capacity and Pi uptake capacity due to low mobility and uneven distribution of Pi in soil [6]. Fine roots are shorter and more with high plasticity [7], and a rapid change in fine root production plays a major role in response to Pi deficiency [8, 9]. The modification of root traits varies significantly across species and cultivars. In *Arabidopsis thaliana* ecotype Col-0, Pi starvation inhibited the primary root growth, but lateral roots and root hairs were increased [10]. Vejchasarn et al. tested 15 rice (*Oryza sativa* L.) genotypes and revealed that Pi deficiency significantly reduced large lateral root density and small and large lateral root length, and increased root hair length and density [11]. Wen et al. characterized root traits of 16 major herbaceous crop species and found crop species with thinner roots displayed a stronger response in root branching, first-order root length, and specific root length of the whole root system in response to limiting soil Pi [12]. The degree of plasticity and relative allocation of root length varied among genotypes. In wheat (*Triticum aestivum*), Pi deficiency modulated the branching distribution of root architecture from linear (evenly spaced branches) to exponential (a greater number of branches at the top of the soil) [13]. Moreover, da Silva et al. reported the Brazilian wheat cultivars with higher P uptake efficiency exhibited total root shallow, enhancing root proliferation in P-rich surface soil [14].

The adaptations to Pi starvation at morphological, biochemical, and physiological levels first contributed to local and systemic sensing and signaling systems [15, 16]. After changes of external Pi concentration, Pi itself, hormones, microRNAs, and sugars serve as signals to elicit transcriptional and posttranslational responses. miR399 is one of the best characterized and important microRNA-mediated P-signaling and well conversed in plants. The miR399 signaling pathway includes several key Pi deficiency responsive genes, *phosphate starvation response 1* (*PHR1*) [17], *induced by phosphate starvation 1* (*IPS1*), *SPX* (named after SYG1/PHO81/XPR1) [18], and *PHOSPHATE 2* (*PHO2*). Among these genes, *IPS1*, a non-protein coding gene, includes a motif with sequence complementarity with miR399 in different plant species [19, 20]. Besides, many hormones serve as Pi signaling components in developmental reprogramming, leading to changes in RSA [16], such as auxin [21], ethylene [22], gibberellin (GA) [23], and jasmonates (JAs). In Arabidopsis, local modifications of auxin concentration induced by low Pi availability involved in the lateral root development [21]; inhibition of ethylene production or action decreased root elongation under Pi deficiency [24]. Transcript levels of genes involved in hormone production, sensitivity, and transport were regulated under Pi stress [25]. The metabolic changes are the ultimate reflection of gene expression. The metabolite profiling could provide a broader view for the final systematic adjustment [26–28]. Metabolomic analysis revealed Pi deficiency led to increases of amino acids, ammonium metabolites, and di- and tri-saccharides, decreases of small P-containing metabolites, and various changes of organic acids in the tricarboxylic acid (TCA) in barley [27], maize [28], common bean [29], and soybean [30]. These changes are the results of more efficient utilization of carbon, nitrogen, and P under low-Pi conditions.

Molecular mechanisms underlying Pi signaling, adaptation and metabolic changes were extensively studied in Arabidopsis and rice [15, 16, 31, 32]. Wheat is one of the most economically important cereal crops in the world. The growth and yield of wheat are limited to low P availability in soil [33]. Thus, P fertilizers are widely and continuously applied in agricultural production system. The consumption of chemical P fertilizers in China had increased approximately 100-fold, from 0.05 Mt in 1960 to 5.3 Mt in 2010 [34]. In addition, the excessive application of P fertilizer causes eutrophication and toxic algal blooms in aquatic ecosystems [35]. The accumulation of P annually in the fields of wheat was 29.4 kg ha^− 1^, which was much higher than those in rice and maize in China [36]. So, it is of great importance to improve low-Pi tolerance and Pi efficiency of wheat for practical food security and agricultural sustainability. Due to the complexity of the hexaploid genome, the related studies in wheat are few and lagging compared with its importance in food crops. The morphological, metabolic, and molecular mechanisms of wheat in response to phosphorus deficiency remained to be elucidated.

China is the world’s largest wheat producer in the last 20 years (110 million metric tonners per year from 1994 to 2016, data obtained from FAO). The average wheat yield in China was steadily increasing from 3.43 t/ha in 1994 to 5.41 t/ha in 2016. It partly contributed to the breeding and popularization of new cultivars with good qualities, stress tolerances, and high yield. Among them, Kenong199 (KN199) was one of the main cultivated wheat cultivars in the northern region of the Huang-Huai winter wheat production base for many years with high nitrogen and P stress resistance and grain yield [37]. In the field experiment, KN199 displayed high tillering capacity, spike formation, and grain production under low P input [38]. However, its underlying mechanism of the low-Pi tolerance of KN199 is still unclear to us. Chinese Spring (CS) exhibited low P uptake, utilization efficiency efficiencies, and grain production under P stress in the field experiment [39]. In this study, we demonstrated the morphological, physiological, metabolic, and molecular characteristics of KN199 in response to Pi deficiency by comparing them with CS. Our results revealed that under Pi deficiency the low-Pi tolerant KN199 had higher Pi and P concentrations in shoots, greater increased root biomass and fine root length, and accumulated higher content of organic acids in TCA cycle and branched chain amino acids in roots than CS. We also provided molecular characteristics for these adaptions for better understanding the mechanisms of low-Pi tolerance in wheat.

## Results

### KN199 was more tolerant to Pi deficiency than CS with significantly increased root biomass and R/S ratio

**Fig. 1:**
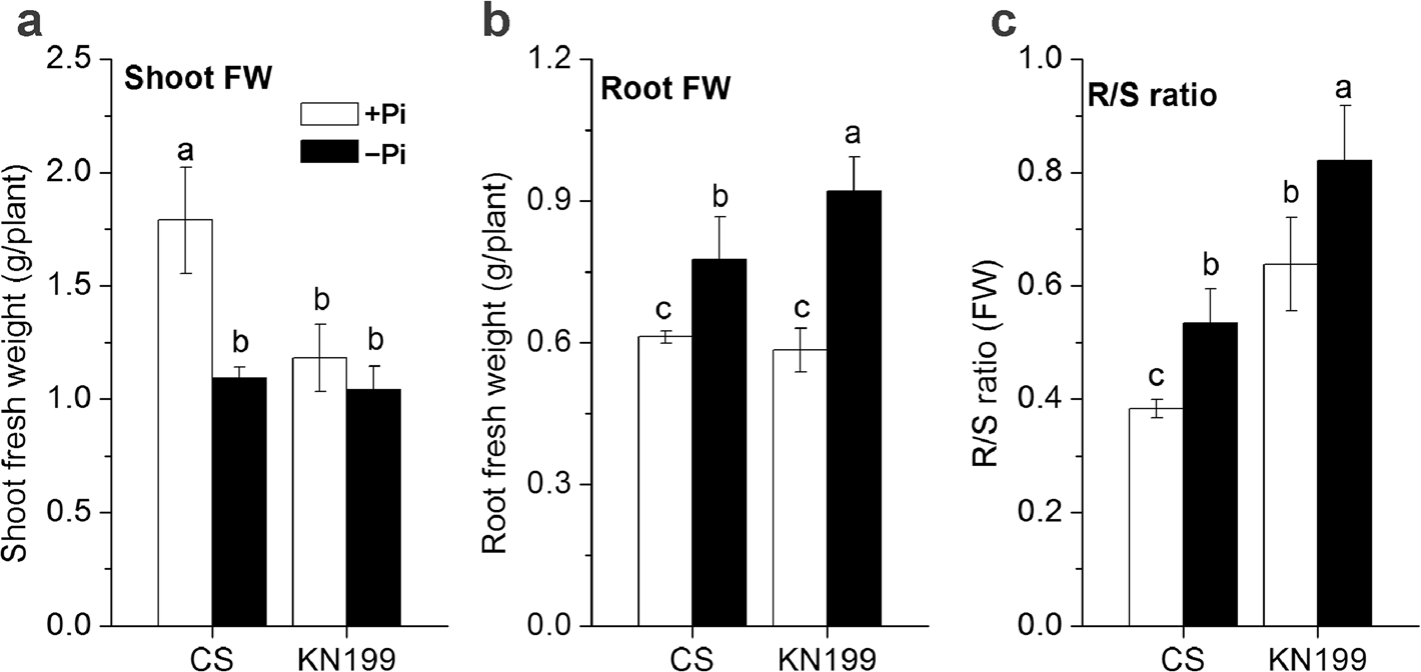
Effect of Pi deficiency on the shoot and root of two wheat cultivars. **a** Shoot fresh weight (FW), **b** root FW, and **c** R/S ratio (FW). Twelve-day-old wheat seedlings were grown on Pi-sufficient (+Pi, white bars) or Pi-deficient (−Pi, black bars) solution for 7 days. Data are means ± SD (*n* = 4). Different lowercase letters indicate significant differences at *p* < 0.05 among columns using the least significant difference multiple range test

To elucidate the low-Pi tolerance mechanism, the effect of Pi deficiency on the two contrasting cultivars, CS and KN199, was investigated at the seedling stage growing under hydroponic condition. The growth of low-Pi sensitive cultivar CS was seriously inhibited after 7 days of Pi deficiency treatment. Compared with Pi-sufficient (+Pi) condition, the shoot fresh weight (FW) of CS was dramatically decreased by 39% under Pi-deficient (−Pi) condition, while the shoot FW of low-Pi tolerant cultivar KN199 was not significantly affected (Fig. 1, a). Conversely, the root FWs of the two cultivars were both significantly increased under Pi deficiency (Fig. 1, b). Moreover, the increase of root FW in KN199 was much higher than that in CS (27 and 58% increase in CS and KN199, respectively). Under −Pi condition, the FW of KN199 was lower than that of CS, so the R/S ratio (FW) of KN199 was higher than that of CS (Fig. 1, c). The R/S ratios were significantly increased by Pi deficiency treatment in both cultivars. Moreover, the R/S ratio of KN199 under Pi deficiency reached the highest value. The root and shoot dry weight (DW) had a similar tendency with these determined by FW (Table 1). The shoot heights of two cultivars were both inhibited by Pi deficiency, but not significantly. Reversely, the root lengths under Pi deficiency were both longer than these under +Pi condition. Remarkably, the increase of root length was significant in KN199, but not in CS (Table 1). In short, compared to the low-Pi sensitive cultivar CS, KN199 was more tolerant to Pi deficiency with drastically increased root biomass and R/S ratio.

**Table 1:** Growth, phosphate uptake, and phosphate utilization efficiency of the two cultivars under Pi deficiency

Traits^a^	Tissues	Chinese Spring (CS)		Kenong199 (KN199)	
		+Pi	−Pi	+Pi	−Pi
Biomass (mg DW^b^/plant)	Shoot	252.7 ± 40.1a	207.4 ± 27.1b	171.2 ± 5.0b	158.9 ± 18.5b
	Root	43.8 ± 5.8bc	49.2 ± 3.6ab	36.9 ± 2.0c	53.3 ± 7.7a
	R/S	0.207 ± 0.017b	0.266 ± 0.010ab	0.236 ± 0.026ab	0.298 ± 0.078a
Length (cm)	Shoot	35.7 ± 1.9a	32.8 ± 2.0a	28.2 ± 2.7b	27.7 ± 3.8b
	Root	32.6 ± 1.9c	33.6 ± 3.7bc	34.9 ± 2.9b	38.9 ± 1.8a
Total P conc.^c^ (mg P/g DW)	Shoot	4.60 ± 0.69b	1.21 ± 0.10d	6.31 ± 0.73a	2.12 ± 0.16c
	Root	6.46 ± 0.15b	2.52 ± 0.26c	7.99 ± 0.37a	2.81 ± 0.13c
Soluble Pi conc. (ug P/g FW^d^)	Shoot	282.40 ± 33.44b	81.31 ± 0.83d	449.72 ± 18.81a	146.07 ± 18.30c
	Root	198.39 ± 36.61b	43.68 ± 6.46c	290.73 ± 40.95a	58.10 ± 10.28c
P content (mg P/plant)	Shoot	1.78 ± 0.22a	0.38 ± 0.03c	1.26 ± 0.27b	0.45 ± 0.88c
	Root	0.55 ± 0.04a	0.18 ± 0.03b	0.50 ± 0.13a	0.20 ± 0.10b
PUE^e^ (g DW/mg P)	Shoot	0.57 ± 0.06c	2.68 ± 0.22a	0.82 ± 0.16c	2.27 ± 0.36b
	Root	1.81 ± 0.14b	5.52 ± 0.78a	2.09 ± 0.48b	4.92 ± 0.28a

Notes: ^a^ 12-day-old wheat seedlings were grown on Pi-sufficient (+Pi) solution or Pi-deficient (−Pi) solutions for 7 days. Data are means ± SD (*n* = 4), and different letters indicate significant differences using the least significant difference multiple range test (*p* < 0.05). ^b^ DW: dry weight; ^c^ conc.: concentration; ^d^ FW: fresh weight; ^e^ PUE: phosphate utilization efficiency

### KN199 had great adaptation in root architecture under Pi deficiency

**Fig. 2:**
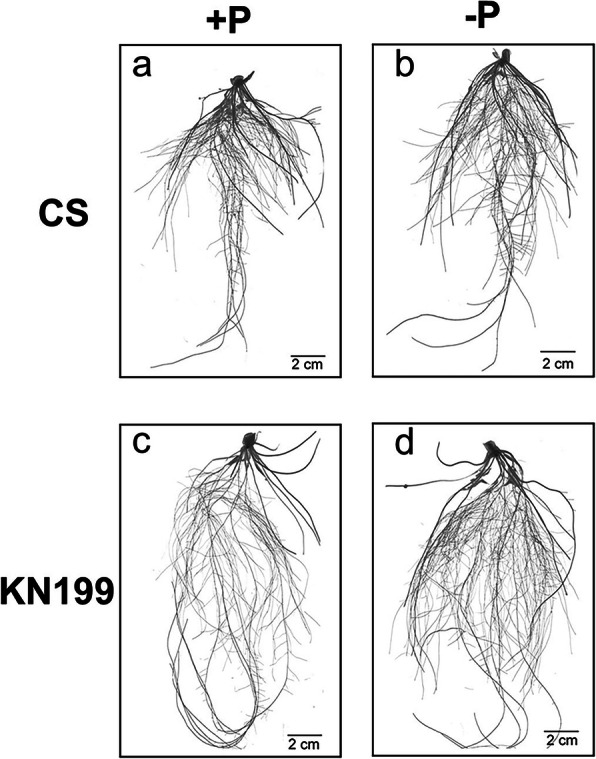
Root morphological performance of the two wheat cultivars. After 7 days’ treatment on Pi-sufficient solution (**a** and **c**) or Pi-deficient solutions (**b** and **d**), photos were taken by the WinRHIZO scanner

For the massive increase of wheat root biomass in response to Pi deficiency, the global root architecture of the two cultivars was further analyzed. Pi deficiency induced the root growth, which was much more apparent in KN199 than that in CS (Fig. 2). After Pi deficiency treatment, the total root length and total root surface area of KN199 significantly increased by 44 and 39%, respectively (Table 2). Fine root represents about 88% of the total root length in two cultivars. Thick root length slightly increased in two cultivars, however not significantly. However, the fine root length of KN199 was significantly increased by 44%, which only 11% in CS. Thus, fine roots contributed to the increase of the total root length of KN199 in response to Pi stress. And the total root volume also increased by 33%. The increases of these traits induced by Pi deficiency in CS were much less than those of KN199, and not significant. The average diameter of roots did not significantly change in the two cultivars. The number of root tips significantly increased in CS under Pi deficiency, but only significantly in KN199. Thus, the increase of root biomass in CS under Pi deficiency was mainly due to the increase of fine roots and root tip numbers; the enormous increase of root biomass in KN199 contributed to the enhancement of total root length (fine roots), total root surface area, and total root volume. Phosphorus is immobile in soils, so the fine root development with longer root length and greater root surface area is crucial for absorbing more Pi, particularly in the low-Pi environments.

**Table 2:** Phenotypic evaluation of global root morphology of the two wheat cultivars under Pi deficiency

Traits^a^	CS		KN199	
	+Pi	−Pi	+Pi	−Pi
Total root length (cm)	361 ± 40b	411 ± 42b	362 ± 25b	522 ± 61a
Fine root length (≤ 0.05 mm in diameter)	313 ± 33b	347 ± 38b	321 ± 28b	461 ± 44a
Thick root length (> 0.05 mm in diameter)	47 ± 8a	63 ± 18a	41 ± 9a	61 ± 17a
Total root surface area (cm^2^)	35.95 ± 5.56b	42.57 ± 7.20ab	37.88 ± 2.55b	52.68 ± 7.75a
Total root volume (cm^3^)	0.285 ± 0.058b	0.300 ± 0.019b	0.316 ± 0.041ab	0.423 ± 0.076a
Average diameter (mm)	0.316 ± 0.017a	0.328 ± 0.033a	0.334 ± 0.025a	0.320 ± 0.012a
Number of root tips	1378 ± 148b	1735 ± 331a	1353 ± 139b	1662 ± 160ab

Note: ^a^ the value for each trait is determined per plant. Data are means ± SD (*n* = 3), and different letters indicate significant differences using the least significant difference multiple range test (*p* < 0.05)

### KN199 showed higher Pi concentrations in shoots than CS under Pi deficiency, but not in roots

The P uptake and PUE in shoots and roots of the two wheat cultivars in response to Pi deficiency were displayed in Table 1. Under +Pi and − Pi conditions, the shoot soluble Pi and total P concentrations of KN199 were all significantly higher than these of CS. Particularly, the shoot soluble Pi concentration in KN199 under −Pi was almost two folds higher than that in CS. But, these variations in roots were small. Under +Pi condition, KN199 still showed significantly higher levels of soluble Pi and total P concentrations in roots than these in CS. While under Pi deficiency, the two cultivars had no significant variations in the root soluble Pi and total P concentrations. For the high biomass of CS under +Pi condition, the shoot P content of KN199 was lower than that of CS. Under Pi deficiency, the shoot P content of KN199 was higher than that of CS. So, KN199 had higher PAE both under +Pi and − Pi conditions. Under limited Pi supply, high Pi level in shoots is a higher priority than that in roots in KN199. Pi deficiency increased the shoot and root PUE (Biomass production per unit of P) in both cultivars. The increase in shoots was more significant in CS (4.7 folds) than that in KN199 (2.8 folds). Besides, the variation in roots between the two cultivars was not significant. Thus, the low-Pi tolerant cultivar KN199 had higher PAE and preferentially maintained high total Pi concentration in shoots in response to Pi deficiency.

### The genes related to miR399-mediated signaling were differentially regulated in shoots, but not in roots

**Fig. 3:**
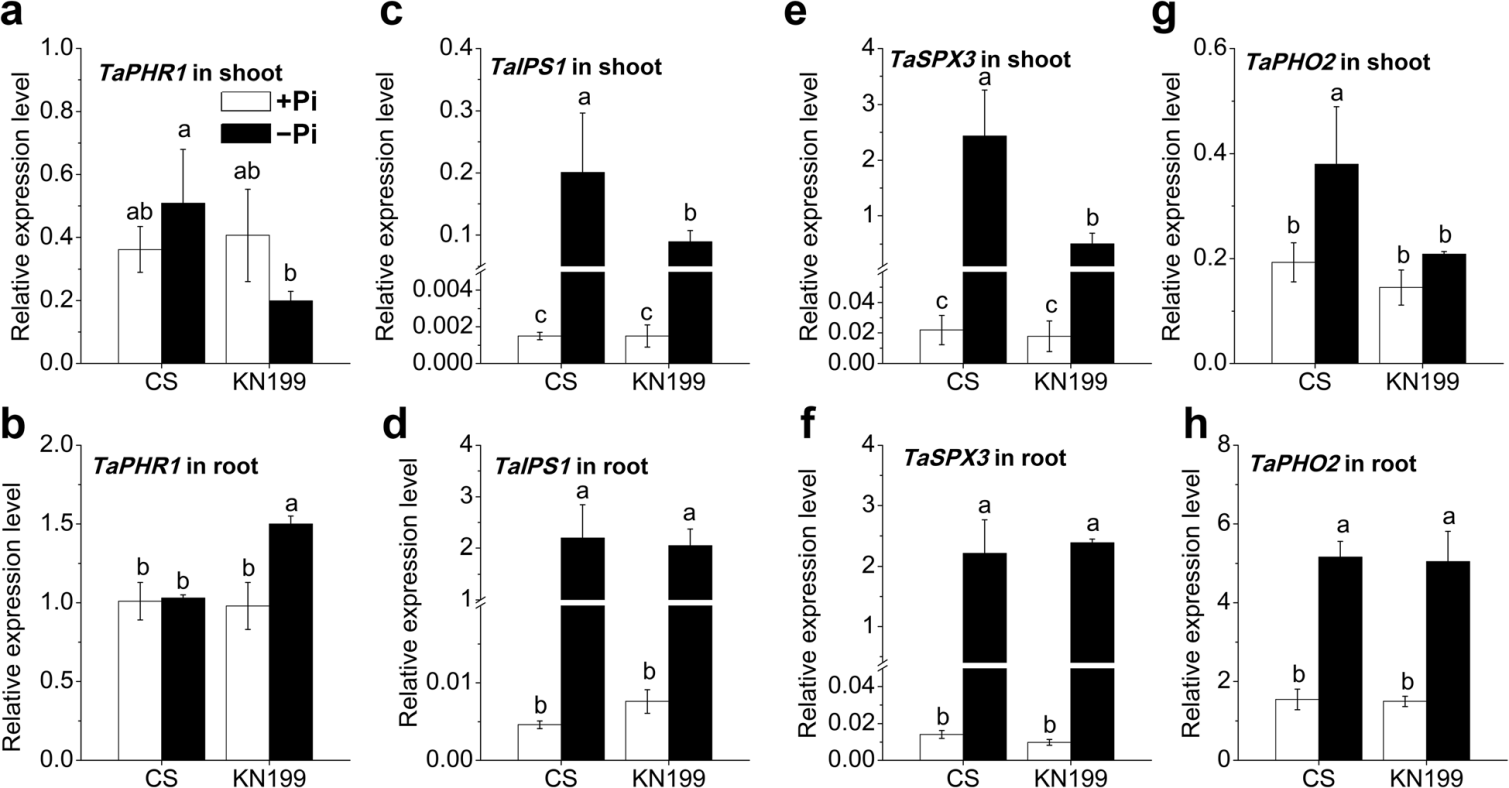
Gene expression of miR399-mediated Pi signaling related genes in response to Pi deficiency. **a-b** *TaPH1*; **c-d** *TaIPS1*; **e-f** *TaSPX3*; **g-h** *TaPHO2* in the shoots and roots of CS and KN199 after 7 days’ treatment on Pi-sufficient (+Pi, white bars) or Pi-deficient (−Pi, black bars) solutions. The gene expression levels were normalized to the internal control of *TaAPT1*. The experiments were done using three biological replicates for transcript analysis with three technical replicates for each cDNA sample. Data represent mean values ± SD (*n* = 3). Columns with different letters indicate significant differences using the least significant difference multiple range test (*p* < 0.05)

To elucidate the molecular mechanism of the favorable adaptations in root traits and P uptake in the low-Pi tolerant cultivar KN199, we first analyzed the gene expression related to the miR399-mediated signaling (PHR1-IPS1-miR399-UBC24/PHO2 signaling cascade), *TaPHR1*, *TaIPS1*, *TaSPX3*, and *TaPHO2* (Fig. 3). PHR1, an MYB-type transcription factor, was a critical factor in regulating the expression of several downstream phosphate starvation responsive genes [40]. Under Pi deficiency, the *TaPHR1* abundance in shoots showed opposite changes between the two cultivars, both not significantly (Fig. 3, a). The *TaPHR1* abundance in the roots was significantly increased by Pi deficiency in KN199, but which did not change in CS (Fig. 3, b). Unlike the small changes of *TaPHR1*, the expression levels of *TaIPS1* and *TaSPX3* in shoots and roots were all dramatically increased by Pi deficiency in the two cultivars (Fig. 3, c-f). Remarkably, the up-regulation in the shoots of KN199 was much lower than these in CS, but they almost had no significant differences between the two cultivars. The expression levels of *TaPHO2* in roots were much higher than those in shoots. Under −Pi condition, the *TaPHO2* abundances in CS significantly increased in shoots and roots, but which in KN199 significantly increased only in roots (Fig. 3, g and h). In short, these key genes related to miR399-mediated signaling were much greater induced in the shoots of CS than those in KN199, while these genes in roots had no significant differential expression between the two cultivars. These differential regulations in shoots between the two cultivars were consistent with the changes in total P and Pi concentration. The Pi deficiency-induced variations in shoot total P and Pi concentrations were larger than that in roots. Under P deficiency, KN199 could maintain higher shoot total P and Pi concentrations than CS. These suggested that the different adaptations in the root architecture of the two cultivars was probably not contributed to the differential regulation of the miR399-mediated signaling pathway.

### The genes related to hormone-mediated signaling were differentially regulated in roots

**Fig. 4:**
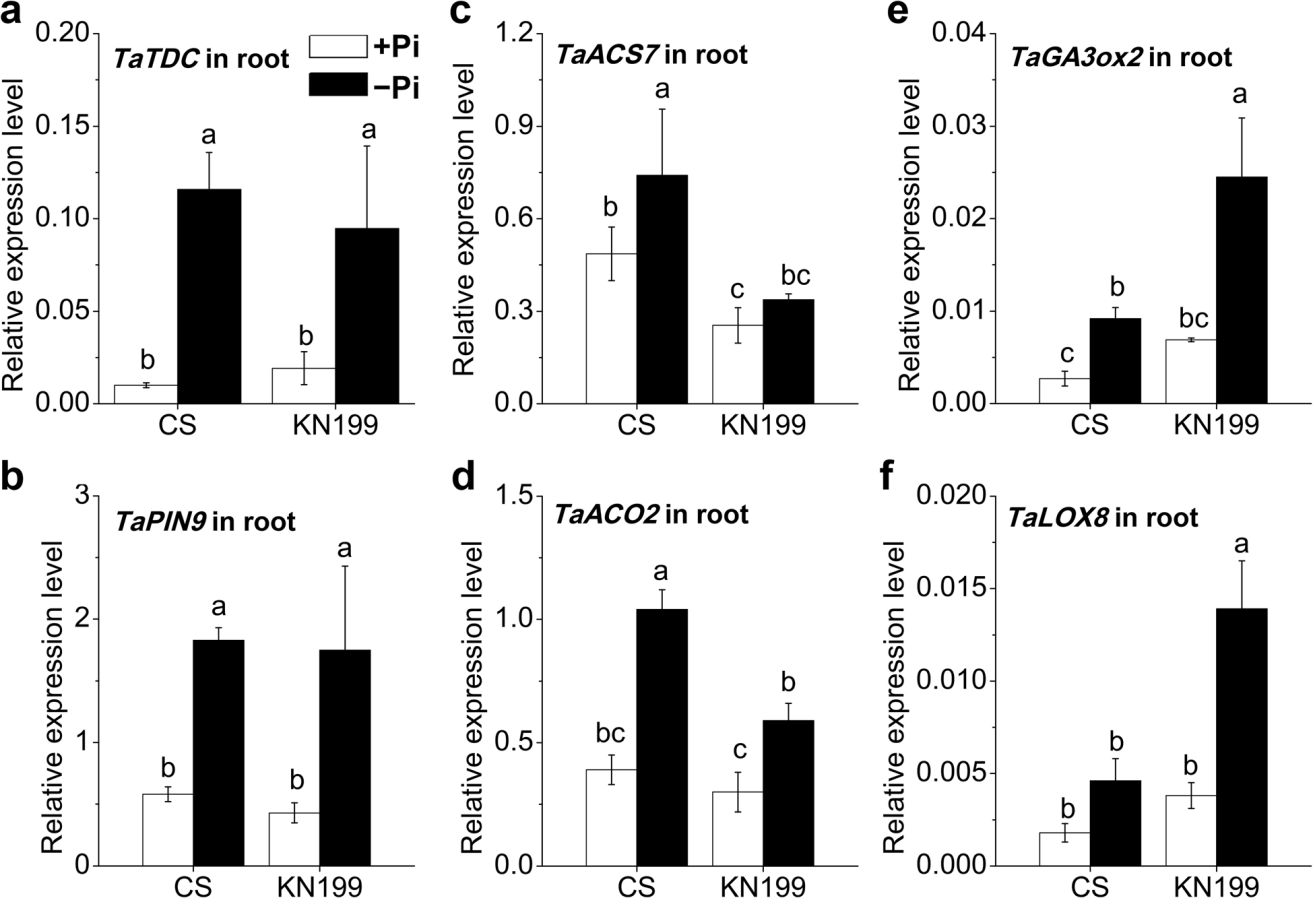
Gene expression of hormone-mediated signaling related genes in response to Pi deficiency. **a** *TaTDC*; **b** *TaPIN9*; **c** *TaACS7*; **d** *TaACO2*; **e** *TaGA3ox2*; **f** *TaLOX8* in the roots of CS and KN199 after 7 days’ treatment on Pi-sufficient (+Pi, white bars) or Pi-deficient (−Pi, black bars) solution. Data represent mean values ± SD (*n* = 3), and different letters indicate differences significantly (*p* < 0.05) using the least significant difference multiple range test

Besides miR399-mediated signaling, many hormones have been reported to involve in Pi starvation signaling pathways, such as auxin, ethylene, GA, and JA. Changes in Pi availability alter hormone production, sensitivity, and transport, and then affect RSA [15, 21]. The transcriptional levels of genes associated with hormone metabolism and transport were tested in the roots of the two cultivars (Fig. 4). Tryptophan decarboxylase (TDC) [41] and PIN-FORMED auxin efflux transporters (PIN) [42] are key proteins involved in auxin biosynthesis and transport, respectively. The *TaTDC* and *TaPIN9* abundances were significantly increased by Pi deficiency in roots (Fig. 4, a and b), however, there was no significant difference between the two cultivars in this regard. The transcript abundances of genes involved in ethylene synthesis, 1-aminocyclopropane-1-carboxylic acid synthase 7 (ACS7) and 1-aminocyclopropane-1-carboxylic acid oxidase 2 (ACO2) [43], were also significantly increased under Pi stress, except *TaACS7* in KN199 (Fig. 4, c and d). The increase of the *TaACS7* abundance in the roots of KN199 was slight. Besides, the up-regulation of *TaACO2* in KN199 was lower than that in CS. Compared with CS, the induction of ethylene biosynthesis by Pi deficiency inhibited in KN199. GA 3-oxidase (GA3ox) and lipoxygenase (LOX) are essential proteins involved in the GA and JA biosynthesis, respectively [44, 45]. The expression levels of *TaGA3ox2* and *TaLOX8* were low in roots. However, the *TaGA3ox2 and TaLOX8* abundances were all significantly induced in the roots of the two cultivars (Fig. 4, e and f). Although these two genes were increased about 2–3 folds by Pi deficiency in the two cultivars, the expression levels in KN199 were about two-fold higher than these in CS. Phosphate deficiency significantly induced the biosynthesis of these hormones in roots, and the genes associated with ethylene, GA, and JA had differential regulation between the two cultivars. These indicated that the hormone-mediated signaling is probably involved in the different changes of the Pi deficiency-induced root traits.

### The genes associated with root development were differentially regulated in roots

**Fig. 5:**
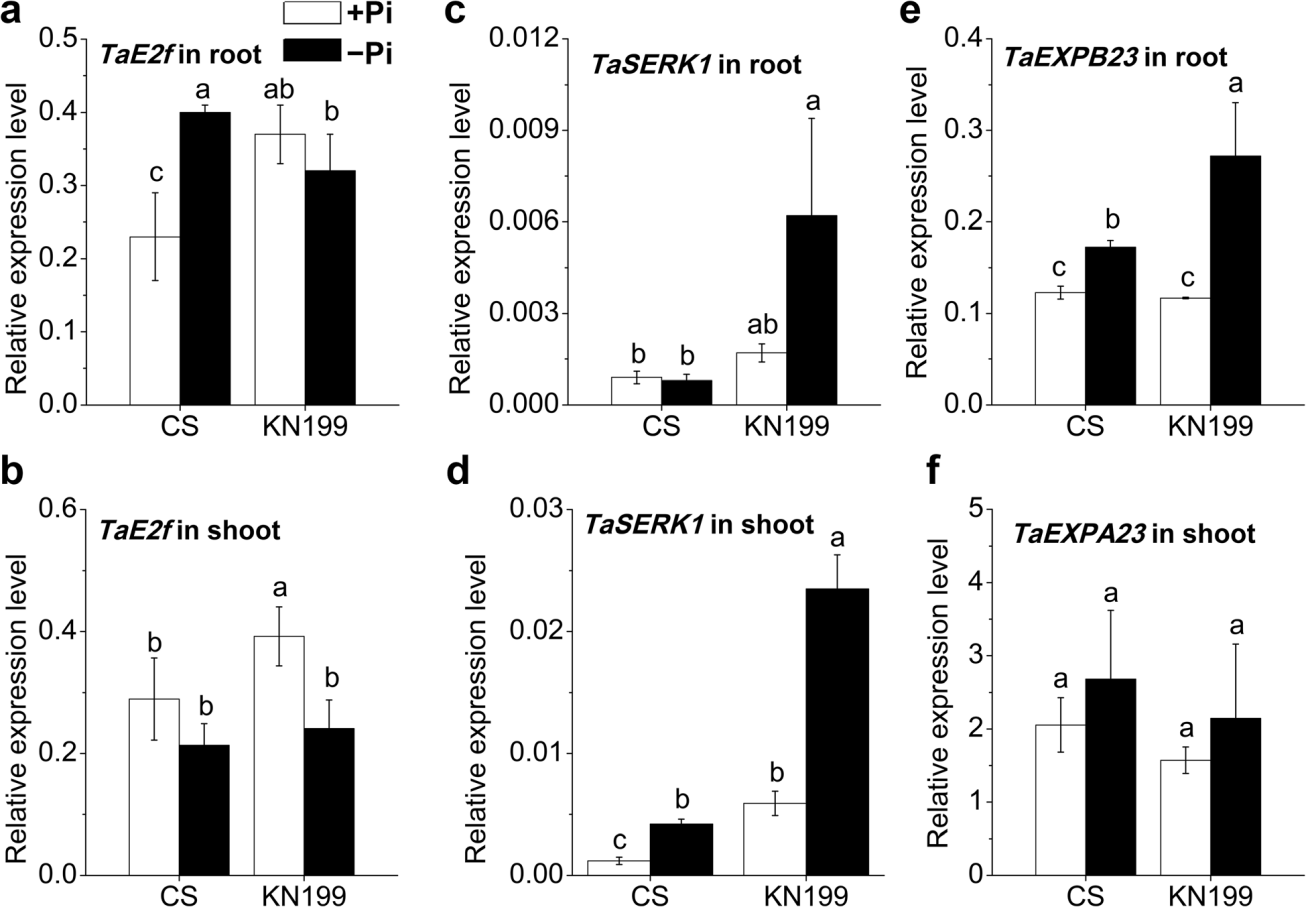
Gene expression of root development related genes in response to Pi deficiency. **a-b** *TaE2f*; **c-d** *TaSERK1*; **e-f** *TaEXPB23* in the roots and shoots of CS and KN199 after 7 days’ treatment on Pi-sufficient (+Pi, white bars) or Pi-deficient (−Pi, black bars) solution. Data represent mean values ± SD (*n* = 3), and different letters indicate significant differences (*p* < 0.05) using the least significant difference multiple range test

Further, the expression of several genes related to root development was determined in the two cultivars. E2F-related (E2F) transcription factors regulated root cell cycle entry and DNA synthesis [46]. A moderate level of *TaE2f* was detected both in roots and shoots of the two cultivars (Fig. 5, a and b). Under Pi deficiency, the *TaE2f* abundance in the roots of CS was significantly increased, while which was slightly decreased in KN199. In shoots, the *TaE2f* abundances were both down-regulated in the two cultivars. The SOMATIC EMBRYOGENESIS RECEPTOR KINASE1 (SERK1) is a member of the Leu-rich repeat, receptor-like kinase protein family, and plays a critical role in root differentiation [47]. The expression levels of *TaSERK1* were relatively low in the roots and shoots of two cultivars (Fig. 5, c and d). Under Pi deficiency, the *TaSERK1* abundances were remarkably up-regulated about 7-folds in roots of KN199, while which had no obvious change in CS. In shoots, the *TaSERK1* abundances were both up-regulated in the two cultivars. And the expression level in KN199 was much higher than that in CS. Expansins are involved in plant development, especially root development [48]. The *TaEXPB23* abundance in roots both increased under Pi deficiency in the two cultivars (Fig. 5, e). The increased fold in KN199 was also much higher than that in CS. The *TaEXPB23* abundances were slightly induced in shoots, and they were no differences between the two cultivars (Fig. 5, f). So, these genes associated with root development were mainly differentially regulated by Pi deficiency between the roots of two cultivars. It was consistent with different Pi deficiency responses in root traits.

### The expression levels of the phosphate transporter 1 (*PHT1*) genes were greatly induced in the roots of KN199

**Fig. 6:**
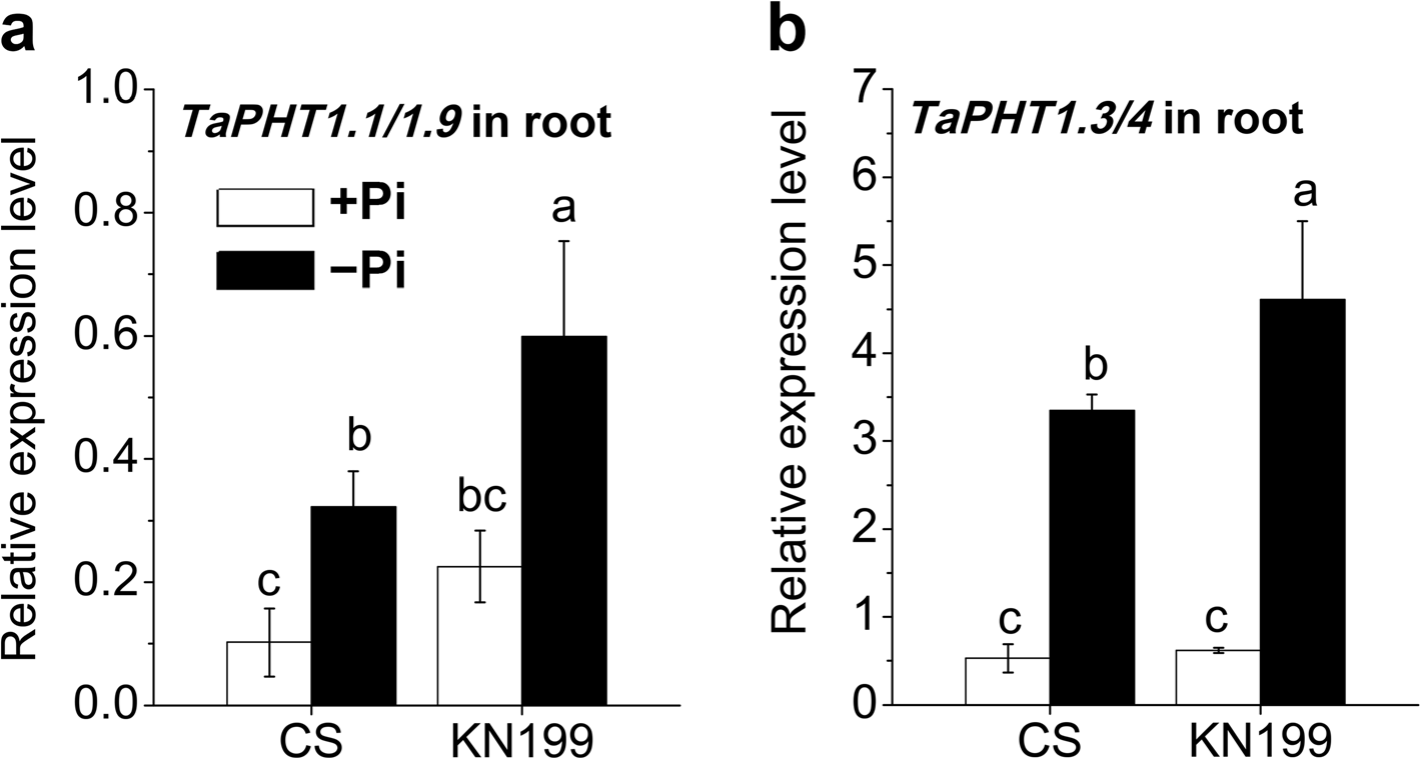
Gene expression of the *TaPHT1s* genes in response to Pi deficiency. *TaPHT1.1/1.9* (**a**) *and TaPHT1.3/4* (**b**) in the roots of CS and KN199 after 7 days’ treatment on Pi-sufficient (+Pi, white bars) or Pi-deficient (−Pi, black bars) solution. Data represent mean values ± SD (*n* = 3), and different letters indicate significant differences (*p* < 0.05) using the least significant difference multiple range test

The *PHT1* family genes mediate Pi uptake and re-mobilization in wheat [49]. We detected the expression levels of *TaPHT1.1/1.9* and *TaPHT1.3* under +Pi and − Pi conditions. The expression of *TaPHT1.1/1.9* gene was root specific. The expression levels of *TaPHT1.1/1.9* were moderate, and which of *TaPHT1.3* were high (Fig. 6, a and b). Under Pi deficiency, the *TaPHT1.1/1.9* and *TaPHT1.3/1.4* abundances were significantly up-regulated in two cultivars. The *TaPHT1.1/1.9* expression level in KN199 was higher than that in CS. Likely, the up-regulation of *TaPHT1.3/1.4* in KN199 was much stronger than that in CS. The higher induction of these *PHT1* genes in the roots of KN199 probably contributed to the uptake of more Pi, maintaining the high level of total P and Pi concentration in shoots under Pi deficiency.

### Accumulations of organic acids and amino acids enhanced in KN199 under Pi deficiency

**Fig. 7:**
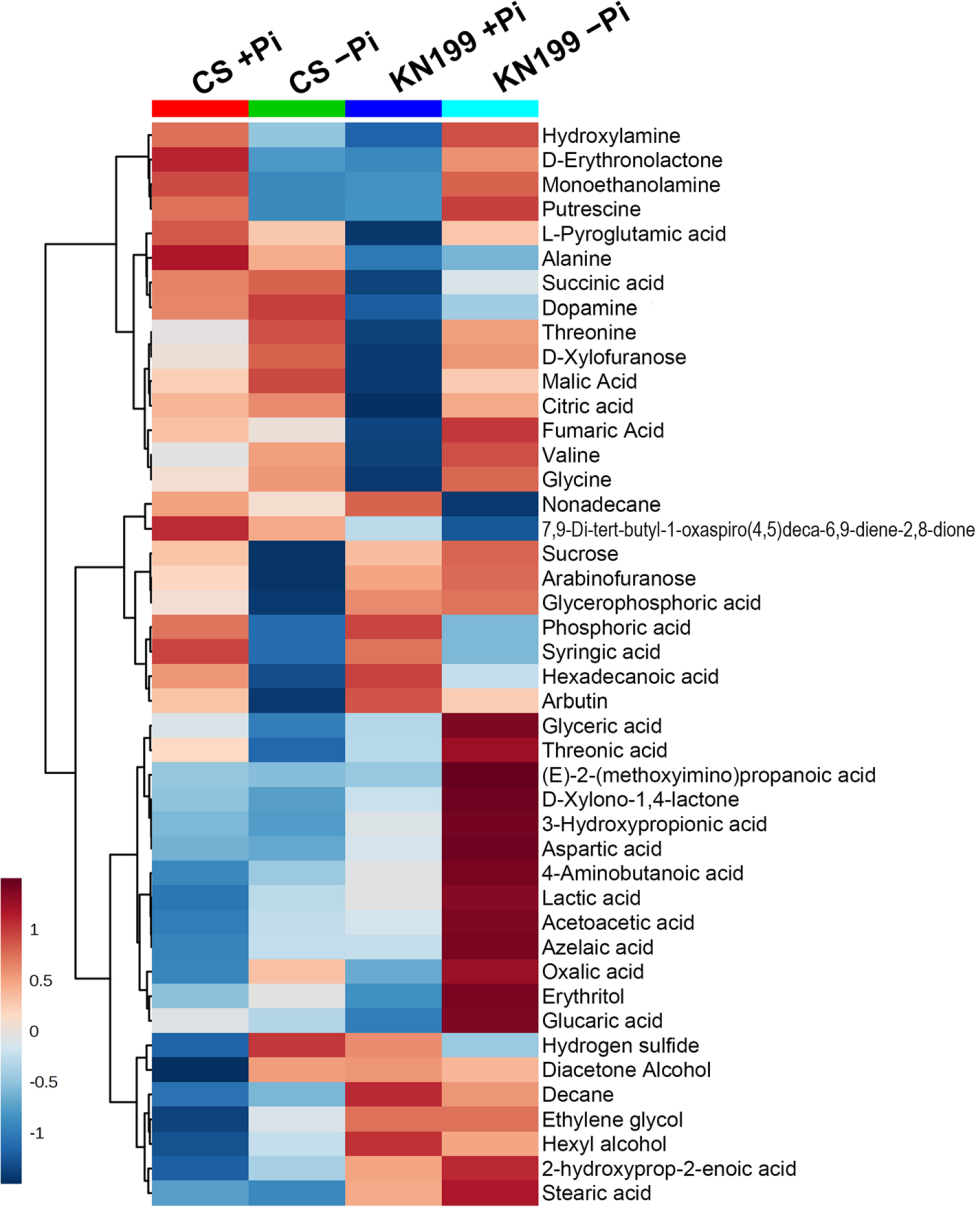
Heatmap and hierarchical cluster analysis for the 44 detected metabolites. The two cultivars, CS and KN199, were detected under Pi-sufficient or Pi-deficient conditions (*n* = 4)

**Fig. 8:**
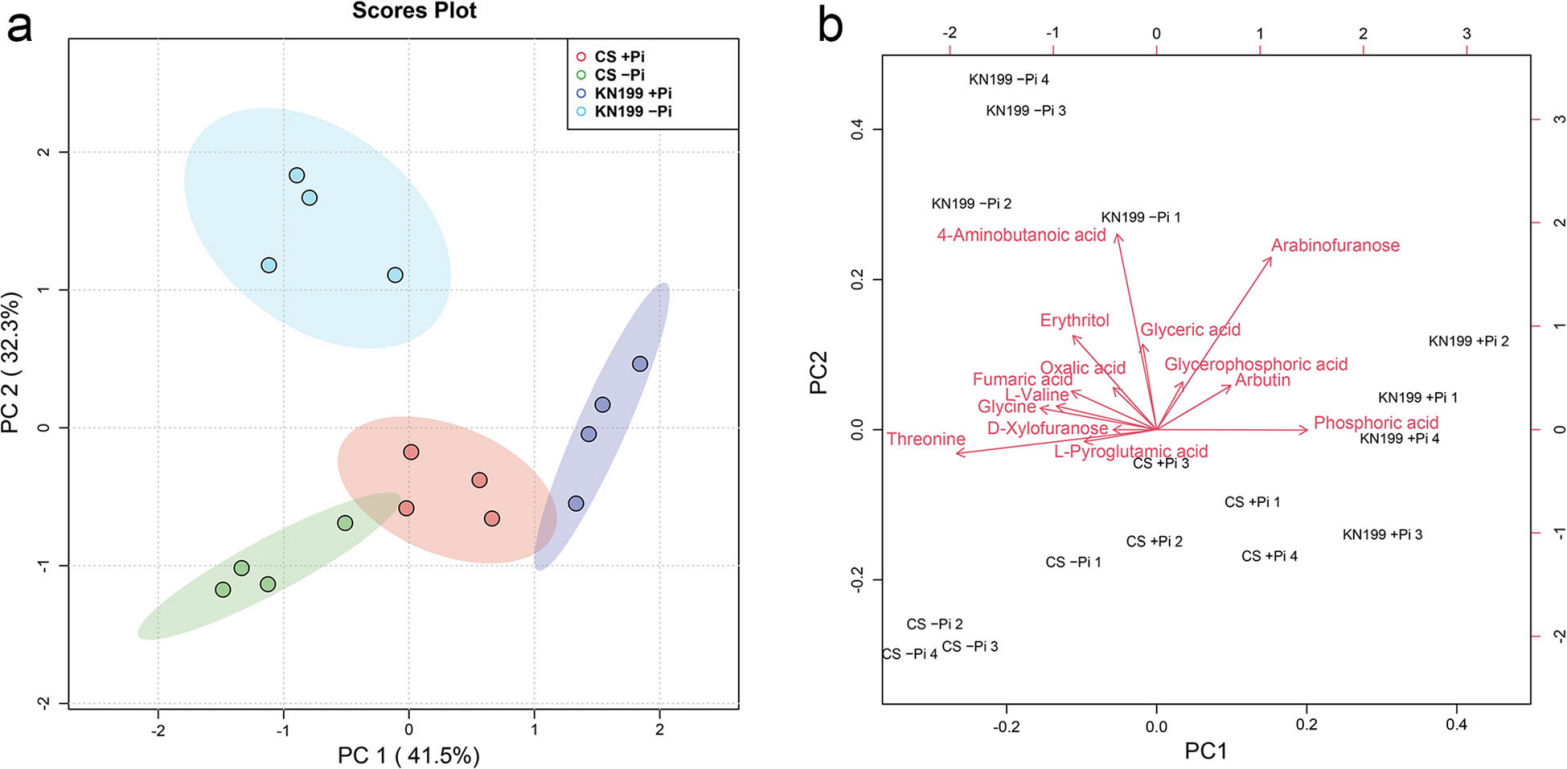
Principle component analysis (PCA) scores plot (**a**) and the corresponding loading plot (**b**) of root metabolome variation among the two cultivars based upon four biological replicates. PC1: the first principal component; PC2: the second principal component. The PCA scores plot distinguishes the metabolic profiles of the two cultivars under +Pi and − Pi conditions. The main metabolites contributing to the PC1 and PC2 are labeled

The metabolic changes in roots were simultaneously investigated using gas chromatography-mass spectrometry (GC-MS). A total of 98 metabolite features were detected in the roots of the two cultivars. However, only 44 metabolites were identified (Additional file 1: Fig. S1 and Additional file 2: Table S1). To find the different performances of the two cultivars in response to Pi deficiency, heatmap analysis of the 44 identified metabolites was conducted. Phosphate starvation changed the abundances of root metabolites, and more remarkable changes were observed in the roots of KN199 (Fig. 7 and Additional file 3: Table S2). Twenty-five metabolites in roots were significantly changed by Pi stress in the two cultivars, including ten organic acids, four amino acids, and 11 other compounds. Principle component analysis (PCA) was performed based on these 25 metabolites (Fig. 8 and Additional file 4: Table S3). PC1 and PC2 accounted for approximately 73.8% of the total variation. Under +Pi condition, the samples of the two cultivars could not separate well, while they separated well under −Pi condition (Fig. 8, a). The samples of KN199 under +Pi and − Pi conditions could separate; however, the samples of CS did not separate. It indicated that the Pi deficiency caused more significant changes in KN199 than CS in root metabolites. The main metabolites contributing to the PC1 included threonine, glycine, L-valine, fumaric acid, etc. (Fig. 8, b).

**Fig. 9:**
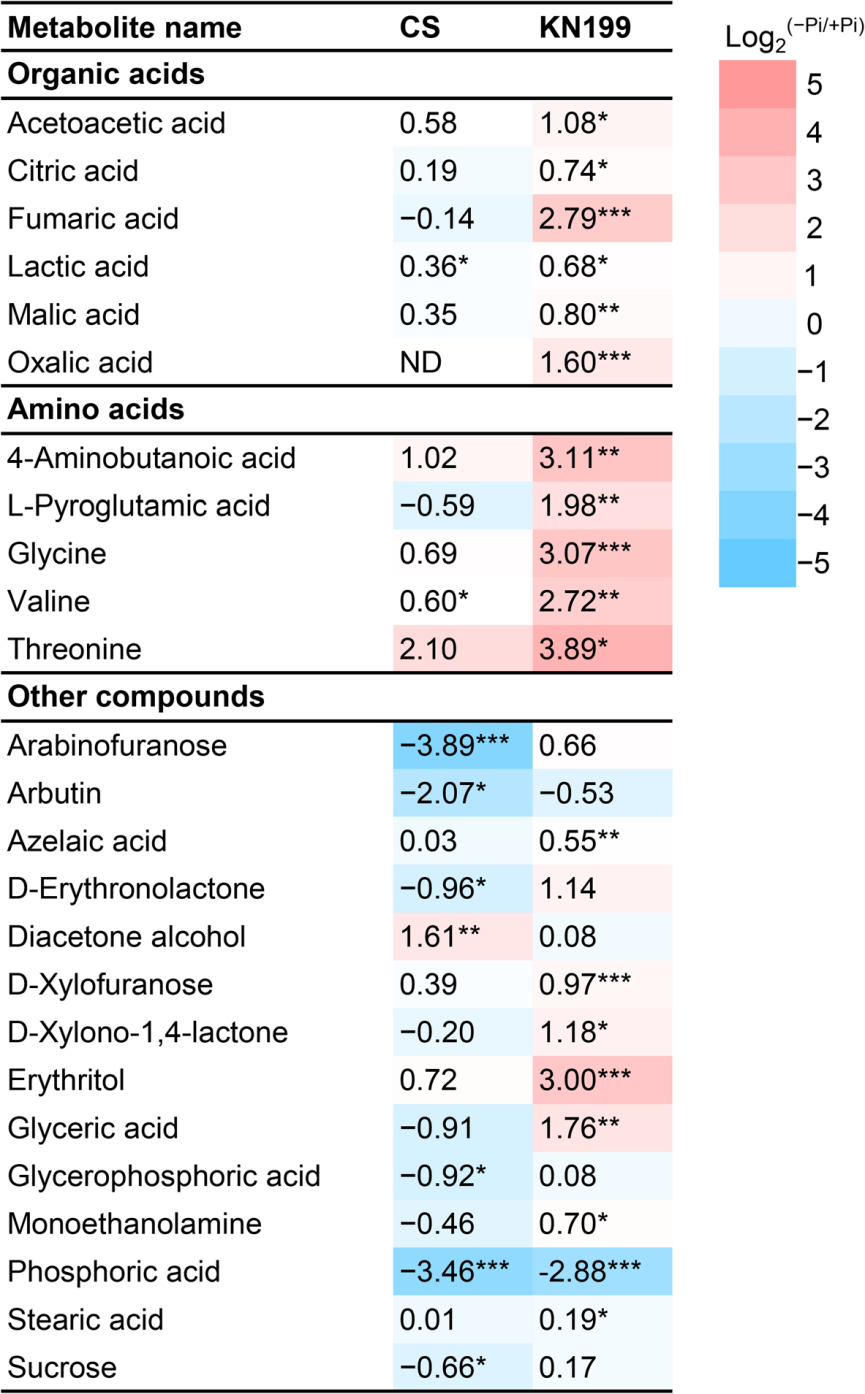
Heat map of metabolite contents in the roots which significant changes in either of cultivar under Pi deficiency. Variations in metabolites are represented by fold change calculated using formula log_2_^(−Pi/+Pi)^. The parts in bold indicate the different categories of metabolites. Asterisks indicate significant differences between P treatments by the student’s *t*-test (*n* = 4), * *p* < 0.05, ** *p* < 0.01, and ***p < 0.001, respectively

Under Pi deficiency, only three and six metabolites in the roots of CS showed significant up-regulated and down-regulated, respectively (Fig. 9). Meanwhile, 18 metabolites significantly accumulated in the roots of KN199. Only the phosphoric acid concentration severely decreased under −Pi condition. Overall, Pi deficiency caused greater increases of metabolites in the roots of KN199 than CS. In particular, the accumulations of amino acids were pronounced. KN199 accumulated higher contents of threonine, glycine, valine, and L-Pyroglutamic acid in response to Pi deficiency (Fig. 9). However, in CS, only the valine concentration showed a significant increase. Similarly, the accumulations of organic acids also significantly increased in the roots of KN199. The concentrations of fumaric acid, oxalic acid, citric acid, malic acid, and lactic acid were increased 1.60–6.93 folds by Pi deficiency. However, the concentrations of these metabolites were not significantly changed in the roots of CS, except lactic acid. Besides, the arabinofuranose, arbutin, D-erythronolactone, glycerophosphoric acid, and sucrose concentrations were significantly decreased by Pi deficiency in CS. While these metabolites did not significantly change in KN199. Thus, the accumulation of primary metabolites, amino acids and organic acids, was remarkably enhanced in KN199 by Pi deficiency.

## Discussion

Pi deficiency causes a severe reduction in crop growth and yield. The development of wheat cultivars with low-Pi tolerance and high-Pi efficiency is thus important for optimal fertilizer use, food security, and agricultural sustainability. The wheat cultivar KN199 showed high stress tolerance and production under low P input in long-term field experiments [38]. Coincidently, KN199 displayed low-Pi tolerance in the seedling stage under hydroponic condition. By comparing with low-Pi sensitive cultivar CS, we revealed the adaption and the relevant molecular mechanisms of low-Pi tolerant cultivar KN199 in this study.

### The low-Pi tolerance of KN199 contributed to great root architecture adaptation

High-yield cultivars were mostly obtained by selecting the above-ground organs, whereas the below-ground roots are generally neglected. Undoubtedly, root traits played vital roles in anchorage, water and nutrient uptake, environmental stress tolerance, and productivity [6]. In our study, the most obvious phenotypic variation in response to Pi deficiency between the two wheat cultivars was the modification in roots. Under Pi deficiency, the total root length (fine roots) and root biomass greatly increased in KN199 with greater total surface area and total root volume. This adaptation in root traits, especially fine roots, was crucial for absorbing more immobile Pi in soil. Fine roots have high plasticity and the potential to change their growth and development to adjust to changing environments [50]. Global analysis of fine root production demonstrated that fine root production also increased with soil P in natural conditions, particularly at P < 300 mg kg^− 1^ [51]. However, its responses differed among plant species, and soil types. In common herbaceous crops, species with thinner roots tended to increase root branching intensity and first-order root length and explore soil P mainly by developing more intensely branched fine roots and higher specific root length [12]. A study based on 215 wheat genotypes demonstrated that genotypes with a greater ratio of seminal lateral root length to seminal axis root length under reduced P condition had a higher root P concentration [52]. Compared to low-P sensitive lines, the tolerant barley lines revealed greater root plasticity in the terms of lateral root length under low-P stress [53]. The adaptation manner in wheat was different from some other plants*.* In *A. thaliana*, the primary root length reduced under the limiting Pi condition, but the formation of lateral roots and root hairs increased [10]. In common bean, total root length and lateral root number greatly reduced under Pi deficiency [54]. The root traits observed in wheat were more similar to those in barley. After 10 days of Pi deficiency, the barely seedlings had more lateral roots, but shorter primary roots [27]. The selection and breeding of high PUE cultivars by root performance was blocked by the difficulty in observing root traits. Ren et al. reported quantitative trait locus pyramiding based on hydroponic culture for root traits provided practical information for development of wheat varieties with large and deep root system and efficient P uptake [55]. However, the effect of Pi deficiency on root development is complex, species-specific, and cultivar dependent [5], breeding of Pi-deficiency-tolerant wheat cultivars is still challenging. The exploration of molecular regulation and key components in response to low Pi response could provide crucial clues for genetic engineering approaches to enhance PAE.

### Molecular regulation for the root adaptation in response to Pi deficiency

The expression of Pi-starvation response genes revealed the possible regulatory mechanisms for the low-Pi tolerance of KN199. The genes related to the miR399-mediated signaling pathway had no significant differences between the roots of two cultivars, while the genes involved in hormones (ethylene, GA, and JA) biosynthesis and root development were differentially expressed between the two cultivars. Ethylene synthesis had implicated in changing the RSA in response to Pi deficiency in plants. In Arabidopsis, inhibition of ethylene production decreased the elongation of roots in low Pi conditions [24]. In common bean, ethylene production per g dry matter in roots was significantly higher under Pi-deficient conditions with reduction of root mass and lateral root density [54]. In wheat, the genes involved in ethylene biosynthesis were significantly increased under Pi deficiency. However, the increase of *TaACS7* was not obvious in KN199 under Pi deficiency. The inhibition of ethylene production may increase the root elongation in KN199. GA stimulated root growth by promoting the destruction of the repressors, a family of nuclear growth-repressing DELLA proteins [56]. In Arabidopsis, Pi starvation caused a decrease in the level of bioactive GA and related genes involved in GA metabolism, triggering the inhibition of root and shoot growth and accumulation of anthocyanin [23]. While in wheat, the *TaGA3ox2* gene involved in GA biosynthesis was both induced in two cultivars, which enhanced the active of GA, and then increased root biomass under Pi deficiency. Transcriptome analyses had demonstrated that genes in hormone biosynthesis and responses were regulated under Pi deficiency [25, 57, 58]. However, evidence for the specificity and direct association of certain hormones needs further studies, particularly in wheat.

Besides, the genes associated with root development, *TaE2f*, *TaSERK1*, and *TaEXPB23*, were also differentially regulated in roots between the two cultivars. In Arabidopsis, transcription factor *E2FC* negatively regulates lateral root formation [46]. The transgenic plant with reduced *E2fc* mRNA levels developed organs with more but smaller cells and increased proliferative activity. The expression regulation of *TaE2f* in response to Pi deficiency was consistent with root morphological changes in the two cultivars. SERKs control somatic embryogenesis and root formation in plants [47, 59]. Overexpression of *TaSERK1* in *Arabidopsis* increased plant height, root length, and even larger silique size and increased seed yield [60]. Expansins may also play roles in root architecture in response to Pi deficiency. The up-regulation of expansin genes in *Stylosanthes* participated in root growth under low Pi condition [61]. *GmEXPB2*, a vegetative β-expansin gene, encodes a secretory protein on the cell wall and is highly induced by Pi starvation in soybean [62]. Overexpressing *GmEXPB2* in *Arabidopsis* increased root cell division and elongation, enhanced plant growth, and P uptake. Consistently, the significantly increased expression of *TaSERK1* and *TaEXPB2* in roots of KN199 contributed to the enhanced root development under Pi deficiency.

### Molecular regulation for the Pi uptake under Pi deficiency

The total P and Pi concentrations could preferentially maintain at the higher level in shoots of KN199 than that in CS. Results showed that the expression of genes involved in the miR399-mediated signaling pathway was differentially expressed in shoots of the two cultivars. miR399 is specifically response to Pi stress as a systemic signal [15, 17]. It directs the cleavage of PHO2 mRNA encoding a ubiquitin conjugating E2 enzyme. PHO2 regulates Pi transport through ubiquitin-mediated protein degradation. An Arabidopsis *pho2* mutant over-accumulated Pi in leaves under Pi stress [17]. The expression level of *TaPHO2* significantly increased in shoots of CS under Pi deficiency, while the abundance of *TaPHO2* in the shoots of KN199 did not significantly change. Thus, the inhibition of *TaPHO2* may contribute to the high Pi concentration in the shoots of KN199. Similarly, the up-regulation of *TaIPS1* and *TaSPX3* was also inhibited in the shoots of KN199. In *A. thaliana*, overexpression of *AtIPS1* increased the accumulation of the miR399 target PHO2 mRNA, resulting in reducing shoot Pi content [19]. In barely, The P-acquisition-efficient cultivar had a low abundance of *IPS1* [20]. These results indicate that low expression of *IPS* genes resulted in highly efficient uptake and Pi accumulation in shoots. SPXs mainly reported as a negative regulator in Arabidopsis and rice in response to Pi stress [63]. In rice, nuclear-localized SPX1 and SPX2 were Pi-dependent inhibitors for the activity of OsPHR2 by inhibiting its binding to the PHR1-binding sequence, P1BS motif [64]. Compared with wide type plants, *Osspx1* and *Osspx2* single mutants had a significantly higher Pi concentration in high Pi conditions. Consistent with the above studies, the high Pi concentration in the shoot of KN199 partly contributed to the weak expression of *TaIPS1* and *TaSPX3*. Besides, the genes *TaPHT1.1/1.9* and *TaPHT1.3* were stronger induced in KN199 under Pi stress, which absorb and transport more Pi. Campos et al. revealed that the higher PAE of the wheat cultivar Crac was associated with an improved *IPS1*-miR399-PHO2 signalling in roots through a fine-tuning modulation of PHO2 activity [65]. In sum, the regulation in miR399-mediated signaling pathway, together with the induction of *TaPHT1* genes, kept KN199 maintain high total P and Pi concentration under Pi deficiency.

### Accumulations of the primary metabolites revealed the accelerated carbon and nitrogen flow under Pi stress

**Fig. 10:**
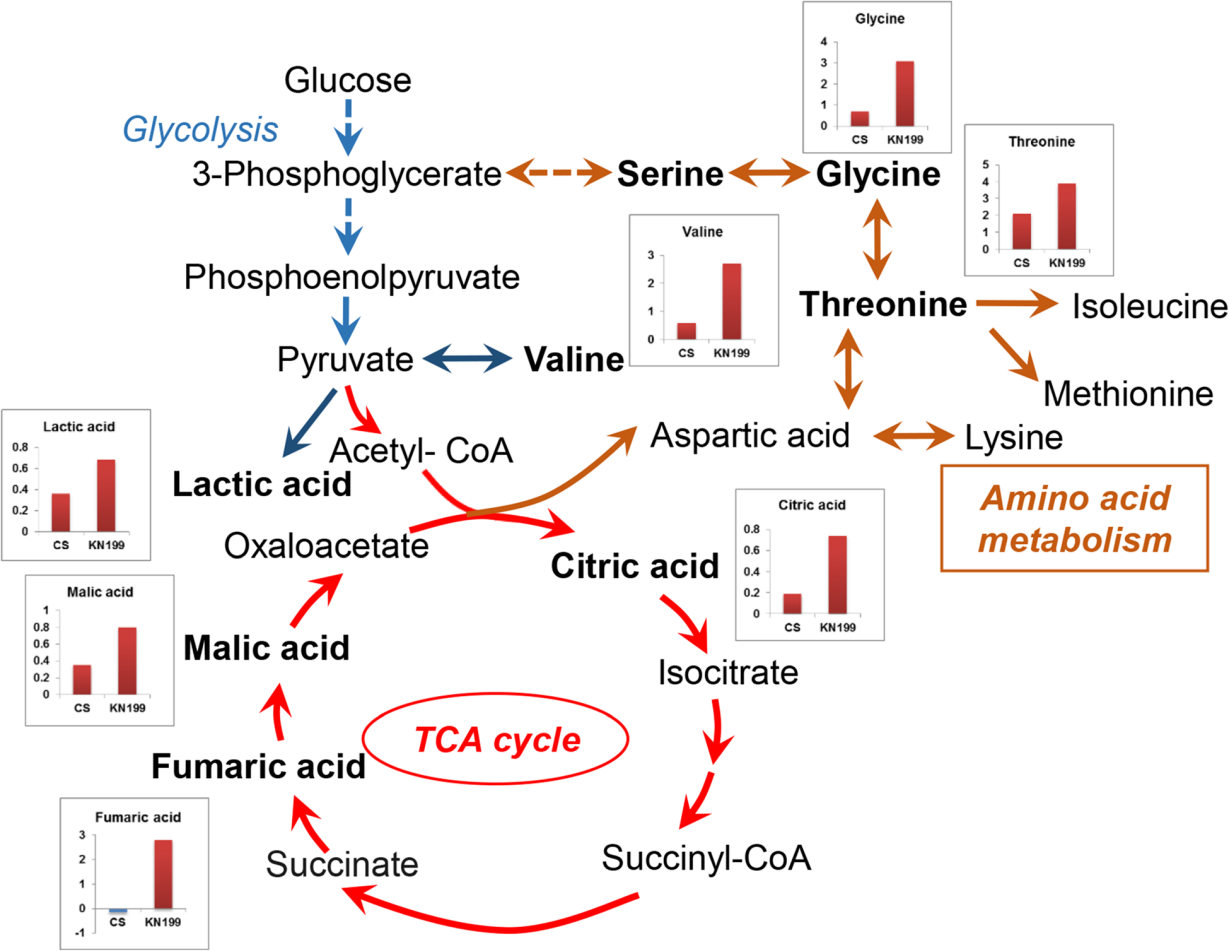
Metabolic pathways of the significantly changed metabolites in response to Pi deficiency. The fold change of the metabolite levels on the Y-axis calculated using formula log_2_^(−Pi/+Pi)^ (*n* = 4). Metabolites with red boxes denote significant increases while with blue ones denote significant decreases. The detected metabolites with significant changes in response to Pi deficiency are highlighted in bold

For in-depth analyzing the changes in roots, nonbiased metabolite profiling using GC-MS revealed that about 56% of identified metabolites differentially accumulated between two wheat cultivars. Most of the primary metabolites detected in the roots increased in response to Pi deficiency. And more remarkable changes were observed in KN199 than these in CS. The primary metabolic adaptations in response to Pi deficiency were in agreement with previous analyses in common bean [29]. Most of amino acids, nitrogen compounds, some organic acids, polyols, and sugars were increased under Pi deficiency, only eight metabolites were decreased in the roots of common bean. Strikingly, organic acids of the TCA cycle were significantly induced under Pi deficiency in the roots of the wheat cultivars, especially in KN199 (Fig. 10). The result was consistent with that observed in the roots of wheat, tomato, chickpea, and white lupin [66]. The malic acid, citric acid, and malonic acid tested in these plants were almost all significantly increased. In barley, the levels of malate, citrate, and α-ketoglutarate were also increased under ten days of Pi deficiency treatment [27]. But after 17 days of Pi deficiency treatment, these organic acids in the barley roots were slightly reduced, indicating the shortage in carbohydrate supply in severely Pi-deficient plants. Some distinct changes were detected in some other plants. In rice, the concentration of lactic acid in roots was increased, while malic acid and citrate were decreased under Pi deficiency [26]. In common bean, different organic acids had different variations. Succinic acid and malic acid were increased and decreased under Pi deficiency, respectively [29]. The abundances of organic acids exhibited large variation between the low-Pi tolerant and low-Pi sensitive maize cultivars [28]. Thus, it appears that metabolic changes in the TCA cycle in response to Pi deficiency differ among plant species and plant cultivars. The increased accumulation of primary metabolites in KN199 indicated that the acceleration of carbon flow in adapt to moderate low-Pi stress of 7 days in wheat.

In many plants, root secrets organic acids into the rhizosphere to enhance Pi solubilization and release fixed P in soils [5]. However, in our study, we tried to collect the root exudates of the two wheat cultivars in response to Pi deficiency. However, the secreted malic acid, lactic acid, acetic acid, citric acid, fumaric acid, and succinic acid were extremely low. Neumann et al. reported the increased secretion of malic acid, citric acid, and malonic acid secreted in chickpea and white lupin, but not in wheat under P stress [66]. In wheat, only malic acid and low concentration of oxalic acid were detected in tested nine organic acids. Our results revealed that little root exudation was induced by Pi deficiency in wheat, although organic acids highly accumulated in the roots.

Several amino acids accumulated in the two wheat cultivars under Pi stress and the increase was also much more pronounced in the Pi-tolerant cultivar KN199 (Fig. 9). Consistent with these observation in some other plants, Pi deficiency induced the accumulation of most amino acids detected in wheat. In the roots of common bean and soybean, most of the amino acids increased under Pi stress, such as threonine, arginine, lysine, and serine [29, 30]. This accumulation of amino acids in roots possibly due to increased protein degradation and repressed protein synthesis under Pi stress. In the roots of severely Pi-deficiency barely, the amino acids involved in ammonium metabolism, glutamine, asparagine, and putrescine, were sharply increased, leading to an increased level of ammonium and alteration in ammonium assimilation [27]. The intracellular accumulation of amino acids could also be used as the source or intermediates of carbon and nitrogen metabolism under Pi deficiency. Especially, valine and threonine, which share the synthetic pathway with branched chain amino acids, displayed the most significant change. These amino acids were common responses under abiotic stresses would be an alternative electron donor for mitochondrial electron transport and an important alternative respiratory substrate [67, 68].

## Conclusions

In summary, our results demonstrated a suite of Pi deficiency responses ranging from root morphology, physiology, gene expression, and metabolites using low-Pi sensitive cultivar CS and low-Pi tolerant cultivar KN199. To adapt to Pi deficiency environment, the KN199 developed more favorable root traits for P uptake, with larger root biomass and longer fine root length. These adaptations probably contributed to the regulation of key genes related to the ethylene, gibberellin, and jasmonates-mediated signaling, root development, and Pi transporters in roots. The total P and Pi concentrations could preferentially keep at a higher level in shoots, which regulated by genes related to miR399-mediated signaling. The organic acids in TCA cycle and branched chain amino acids also accumulated as the preferred storage metabolites in the roots of KN199 for enhanced carbon and nitrogen metabolism. The understanding of mechanisms through the two contrasting wheat cultivars (the wild and cultivated variety) would help to design more effective breeding and genetic engineering strategies to produce highly PAE crop.

## Methods

### Wheat cultivars and growth conditions

Bread wheat (*Triticum aestivum* L.) cultivar KN199 and CS were used in this study. Seeds of these two cultivars were provided by Professor Xiuying Kong from Chinese Academy of Agricultural Sciences, China. CS was the model wheat, which widely studied [69]. KN199 was derived from a cross between Kn9204 (a 1RS/1BL translocated and derivative of wheat *Thinopyrum ponticum* partial amphiploid) and Shi4185 (developed by Institute of Genetics and Developmental Biology, Chinese Academy of Sciences) [37], and has been commercialized now. No special permissions are necessary to collect and use plant materials. All the plant materials were grown in compliance with the legislation of China.

The healthy seeds of two cultivars (CS and KN199) were surface-sterilized with 10% H_2_O_2_ and then germinated on filter paper saturated with distilled water in darkness at 25 °C for 1 day. The seeds were transplanted into a plastic net suspended on a plastic pot (1.25 L) filled with 0.5 mM CaCl_2_ solution (pH 4.5) for 3 days. And then, the seedlings with similar growth parameters were selected and transplanted into 1/4 modified Hoagland solution. Each 1.25 L black pot had five seedlings. To study the Pi deficiency response, the 12-day-old seedlings were grown on Pi-sufficient (250 μM Pi, +Pi) or Pi-deficient (0 μM Pi, −Pi) 1/4 modified Hoagland nutrient solution for 7 days [70]. The Pi-sufficient nutrient solution contained 250 μM KH_2_PO_4_, and the Pi-deficient nutrient solution did not contain KH_2_PO_4_ with an additional 250 μM KCl. The modified 1/4 Hoagland solution was composed of (μM): Ca (NO_3_)_2_·4H_2_O (2000), MgSO_4_·7H_2_O (650), KH_2_PO_4_ (250), K_2_SO_4_ (750), KCl (100), MnSO_4_. H_2_O (10), CuSO_4_·5H_2_O (0.1), ZnSO_4_·7H_2_O (1), H_3_BO_3_ (1), (NH_4_)_6_MoO_24_·4H_2_O (0.05), and Fe (III)-EDTA (40) [70]. The pH was adjusted to 6.0. The solutions were replenished regularly at three-day intervals. The hydroponic experiment was performed at the Institute of Soil Science, Chinese Academy of Sciences, Nanjing, China. The seedlings were grown in a temperature-controlled growth chamber with a 14-h/26 °C day and a 10-h/23 °C night cycle, a light intensity of 300 μmol m^− 2^ s^− 1^, and a relative humidity of 60%. At least three biological replicates were performed for each treatment and sampling.

### Soluble Pi concentration and total P concentration

Shoots and roots of seedling were sampled respectively, weighed, and homogenized in liquid nitrogen to determine the soluble Pi concentration. Phosphate was extracted with 8 mL of 5% (v/v) sulphuric acid (5 M) solution. The supernatants were obtained by centrifugation at 12000 *g* for 15 min for Pi determination. To determine the total P concentration, the oven dried samples were ground to powders and digested with 5 mL H_2_SO_4_ for 3 h at 150 °C. Meantime, H_2_O_2_ was added to make the samples properly digest. The total P and soluble Pi concentrations were measured using the molybdate blue-colorimetric method [71]. The diluted samples (pH adjusted) were mixed with 15% (w/v) fresh ascorbic acid dissolved in ammonium molybdate (pH 5.0) and then incubated at 37 °C for 30 min. The absorbance at 650 nm was recorded. The Pi and total P concentrations were calculated using FW and DW normalization, respectively.

### Global root morphology analysis

To characterize the global root morphology, the harvested roots were dispersed on a clear perspex tray with water and scanned using a root system scanner (EPSON Expression 1600, Seiko EPSON Corp., Japan). The images were analyzed using WinRHIZO software (WIN MAC, Regent Instruments Inc., Canada; http://www.regentinstruments.com/). WinRHIZO output included total root length per plant in centimeter (cm), fine root length (≤ 0.5 mm in diameter), thick root length (> 0.5 mm in diameter) [7], total root surface area in cm^2^, average diameter in millimeter (mm), total root volume per plant in cm^3^, and the number of tips.

### Gene expression analysis

Total RNA was extracted from homogenized frozen shoots or roots (50 mg) according to the operations manual supplied by Plant RNA Kit (TianGen, OSR-M610) and convert into cDNAs using Prime Script reverse transcriptase (Toyobo, FSQ-101). The quality and quantity of RNA were assessed using agarose gel electrophoresis and the NanoDrop 2000 (Thermo Fisher Scientific, USA). The quantitative real-time PCR reactions were performed in a 10 μL reaction volume containing 0.01 μg of cDNA, 0.2 μM of each primer, and 5.0 μL of SYBR Green Realtime PCR Master Mix (Toyobo, QPK-201). Three technical replicates were conducted for each cDNA sample. Three biological replicates were used for transcript analysis. The wheat adenine phosphoribosyltransferase (*TaAPT1*) gene was used as housekeeping gene [70]. The relative expression levels of genes were calculated according to the 2 -^△△^Ct method [72]. The primer sequences for the tested genes are listed in Additional file 5: Table S4.

### GC-MS analysis of intracellular metabolites in roots

The metabolite extraction and derivatization were performed as described by Ganie et al. [28]. The homogenized frozen roots (100 mg) were extracted with pre-cooled solvents, isopropanol: acetonitrile: water (3:3:2) and chloroform: water (1:2). The methanol/water phase was dried by vacuum centrifugation (Concentrator Plus, Eppendorf, Germany) overnight. After carbonyl moieties were protected by methoximation, the acidic protons were derivatized with 70 μL N-methyl-N-trimethylsilyl trifluoroacetamide (Sigma-Aldrich Co., USA) at 37 °C for 90 min. The trimethylsilyl samples were separated and analyzed using the GC-MS system (GC: Varian CP-3800, MS: Saturn 2200, USA) according to the method of Ganie [28]. Mass spectra were recorded from m/z 35 to 600 for trimethylsilyl derivatization. Compounds were identified by comparing them with the reference compounds in MainLib or Wiley Registry8e (Additional file 1: Fig. S1 and Additional file 2: Table S1). Mass spectral matching was manually supervised, and matches were accepted with thresholds of match 700. The areas of the identified metabolites were analyzed using MetaboAnalyst 4.0 online (https://www.metaboanalyst.ca/MetaboAnalyst/faces/home.xhtml) [73]. The remaining missing values were estimated using the Singular Value Decomposition method [74]. Log normalization was used for data transformation. Features were removed with > 51% missing values. The identified metabolites were comprehensively compared using the PCA and Heatmap analysis. The fold-change of metabolite between control and treatment was transformed by a logarithmic base of 2.

### Statistical analysis

Data represent means ± SD of at least three biological replicates. Student’ t-test or two-way analysis of variance (ANOVA) was conducted between two cultivars under +Pi and − Pi conditions and followed by the least significant difference (LSD) multiple range test (*p* < 0.05) using IBM SPSS statistical software (version 22.0.0.0) for statistical analysis.

## Supplementary Information

**Additional file 1.**

**Additional file 2.**

**Additional file 3.**

**Additional file 4.**

**Additional file 5.**

## Data Availability

All data generated or analyzed during this study are included in this published article and its supplementary information files.

## Abbreviations

+Pi/−Pi: Sufficient/deficient-inorganic phosphate
ACO2: 1-Aminocyclopropane-1-carboxylic acid oxidase 2
ACS7: 1-Aminocyclopropane-1-carboxylic acid synthase 7
DW: Dry weight
E2F: E2F-related
FW: Fresh weight
GA: Gibberellin
GA3ox: GA 3-oxidase
GC-MS: Gas chromatography-mass spectrometry
JAs: Jasmonates
LOX: Lipoxygenase
P: Phosphorus
PAE: Phosphate acquisition efficiency
PCA: Principle component analysis
Pi: inorganic phosphate
PHO2: PHOSPHATE2
PHR1: Phosphate starvation response 1
PUE: Phosphate utilization efficiency
RSA: Root system architecture
SERK1: SOMATIC EMBRYOGENESIS RECEPTOR KINASE1
TCA: Tricarboxylic acid
