# Supplementary material for: Greater morphological and primary metabolic adaptations in roots contribute to phosphate-deficiency tolerance in the bread wheat cultivar Kenong199
Source: BMC Plant Biol. 2021 Aug 19;21:381. doi: 10.1186/s12870-021-03164-6 (PMC8375062; doi:10.1186/s12870-021-03164-6)
Supplement: Supplementary file 1 — Additional file 1. [file 12870_2021_3164_MOESM1_ESM.docx]

**Additional file 1: Figure S1** Mass spectrum of the identified metabolites using GC-MS method

1. Hydrogen sulfide


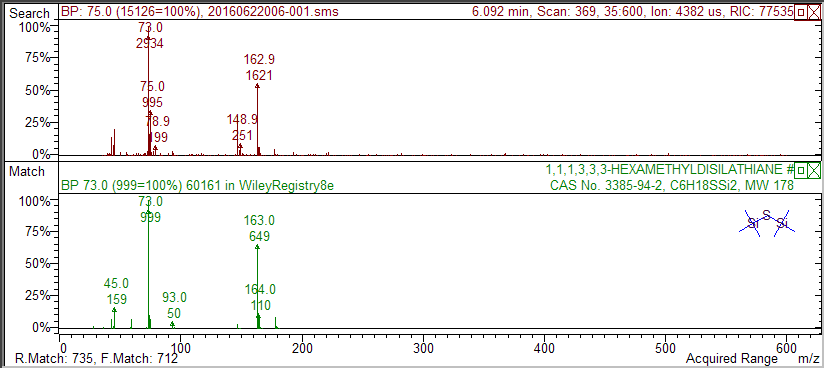


2. Acetoacetic acid


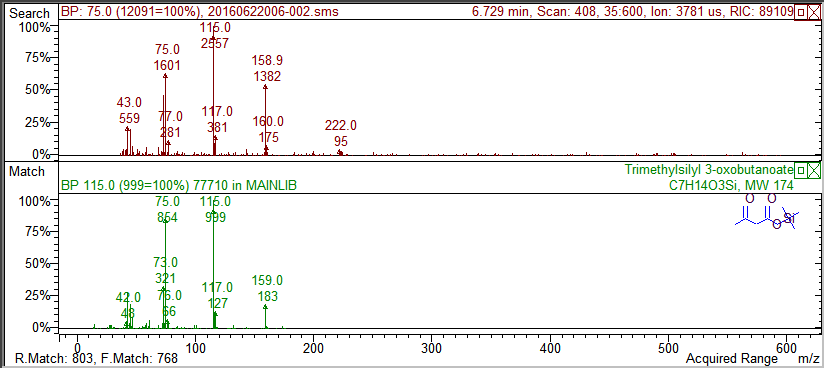


3. Ethylene glycol


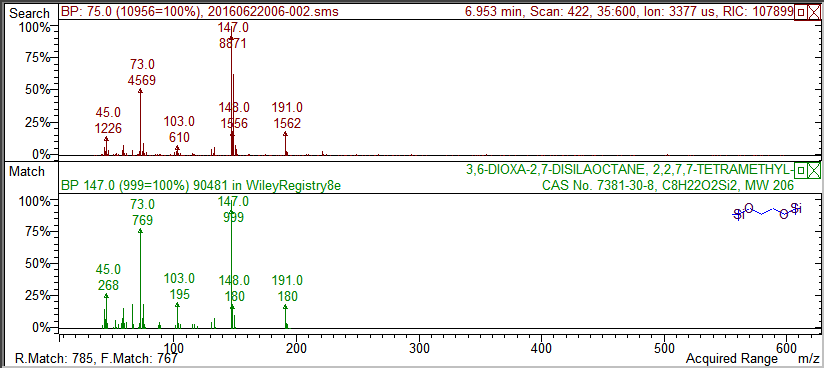


4. Hexyl alcohol


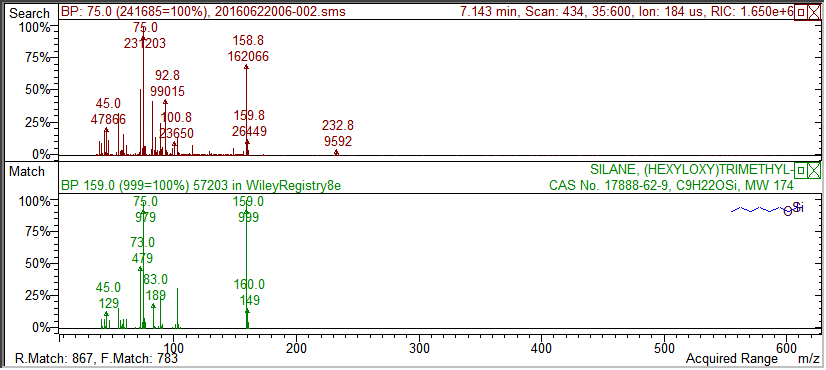


5. Diacetone alcohol


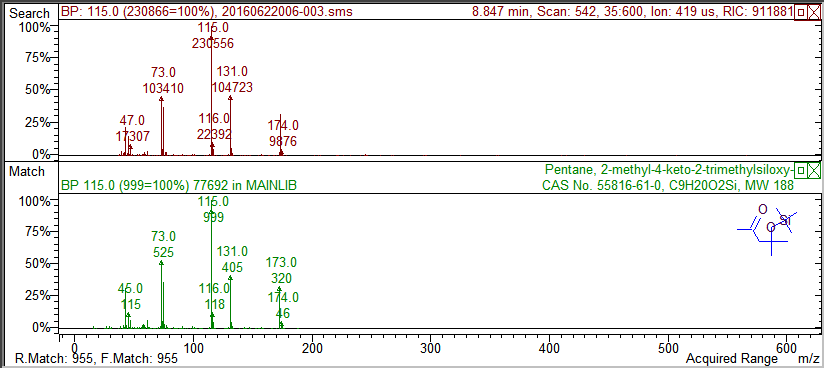


6. (2E)-2-(Methoxyimino)propanoic acid


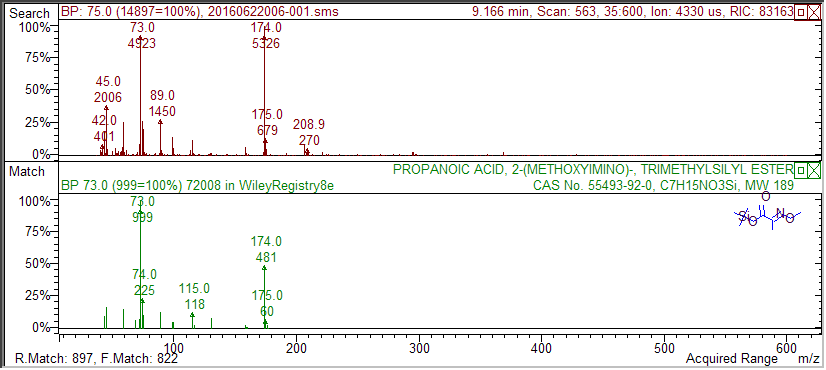


7. Lactic acid


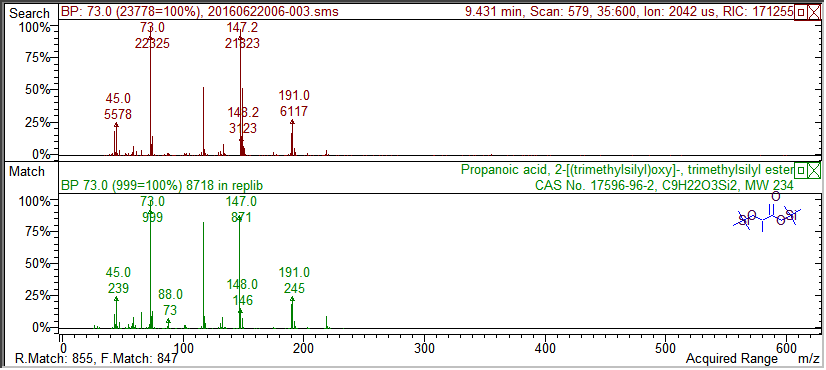


8. 2-Hydroxyprop-2-enoic acid

**
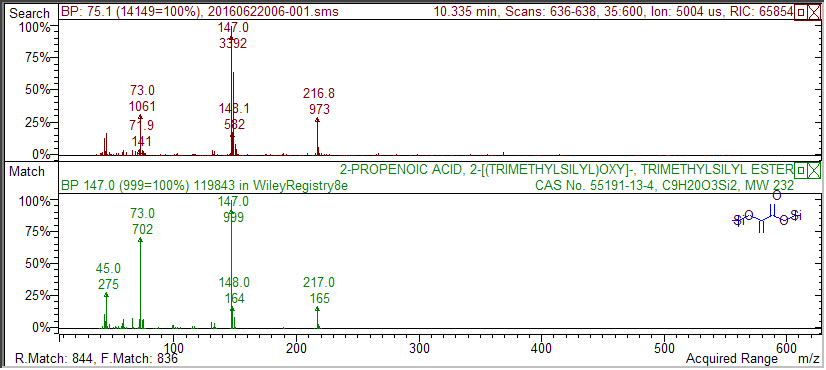
**

9. Alanine


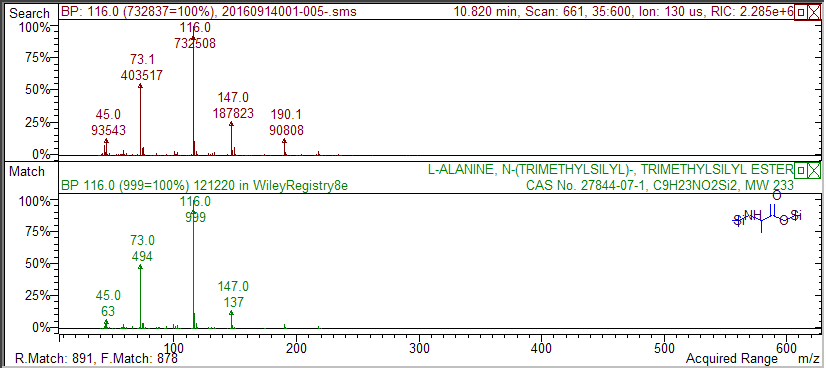


10. Hydroxylamine


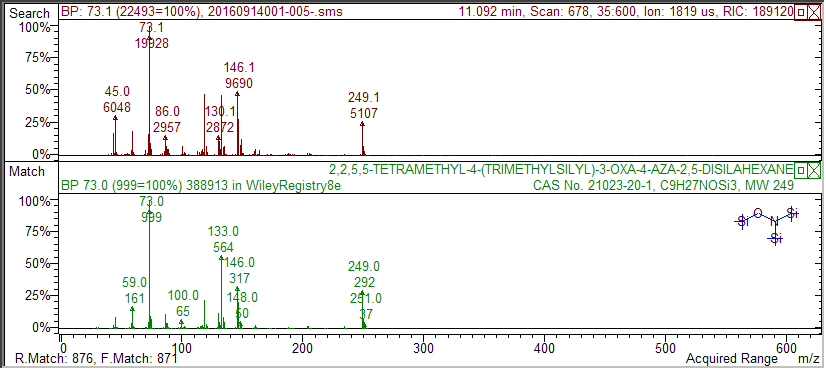


11. Oxalic acid


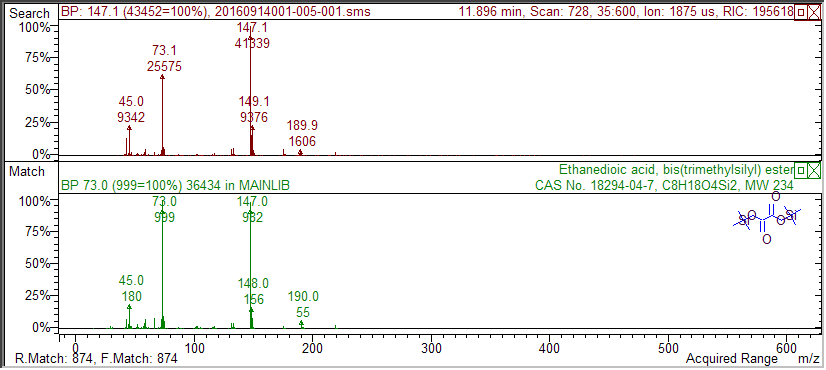


12. 3-Hydroxypropionic acid

**
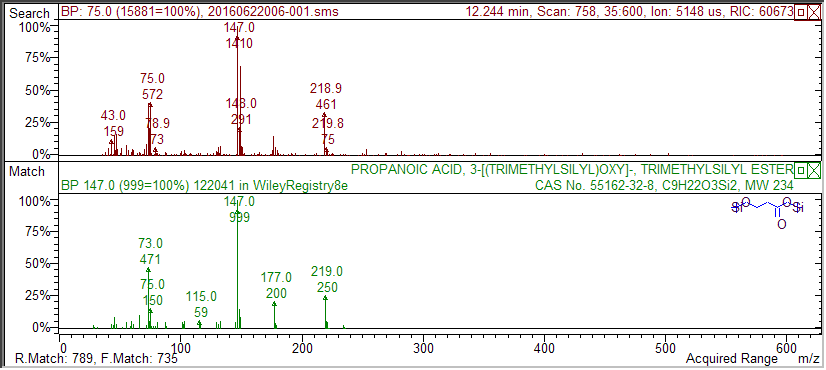
**

13. L-Valine


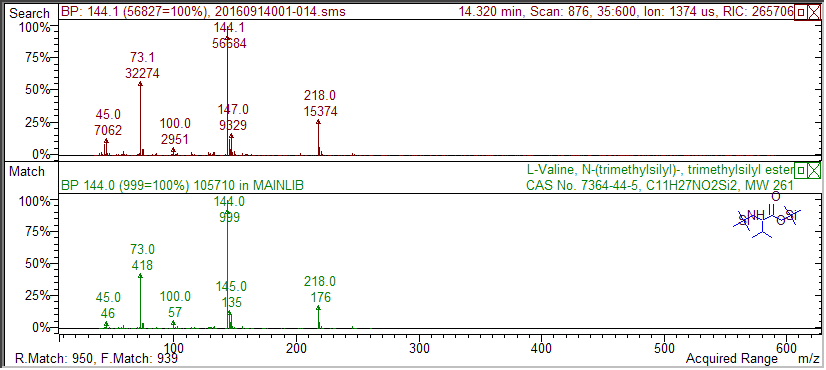


14. Monoethanolamine

**
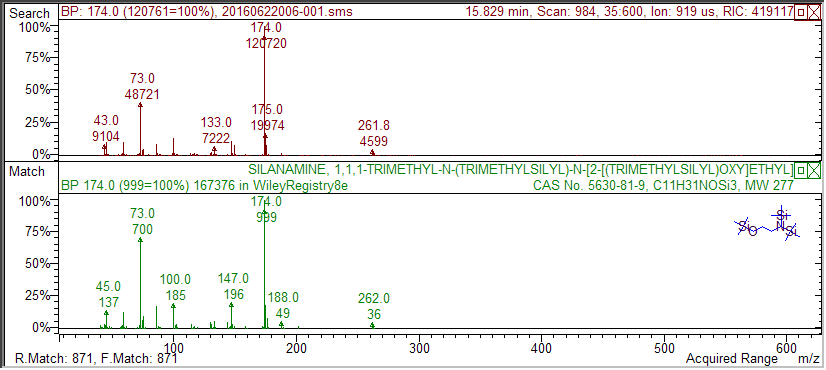
**

15. Phosphoric acid

**
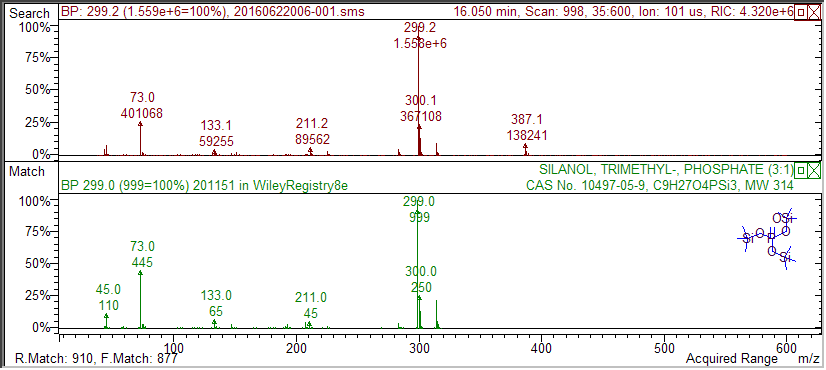
**

16. Glycine


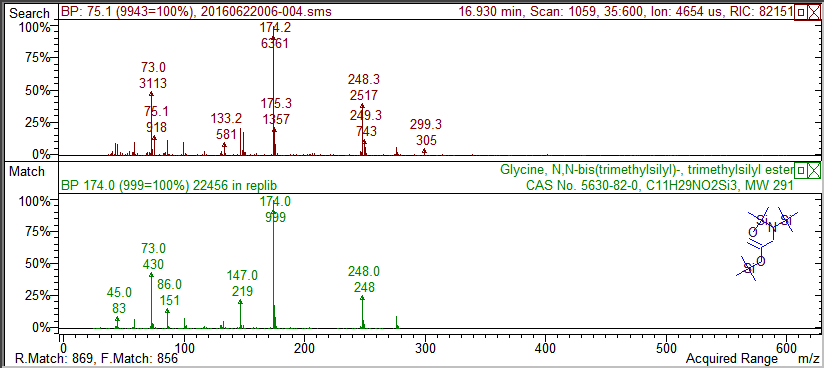


17. Succinic acid


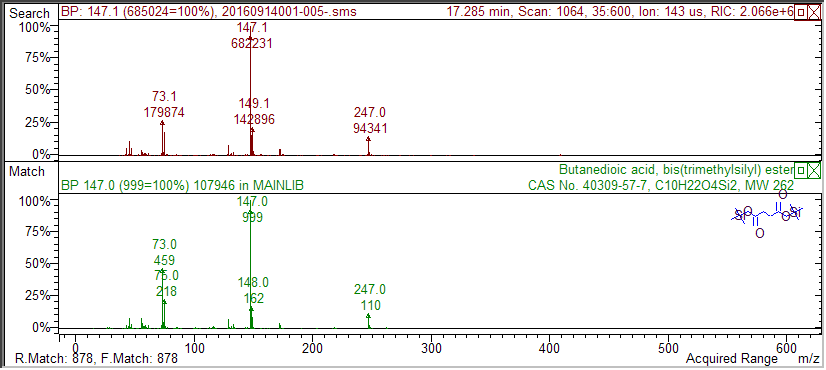


18. Decane

**
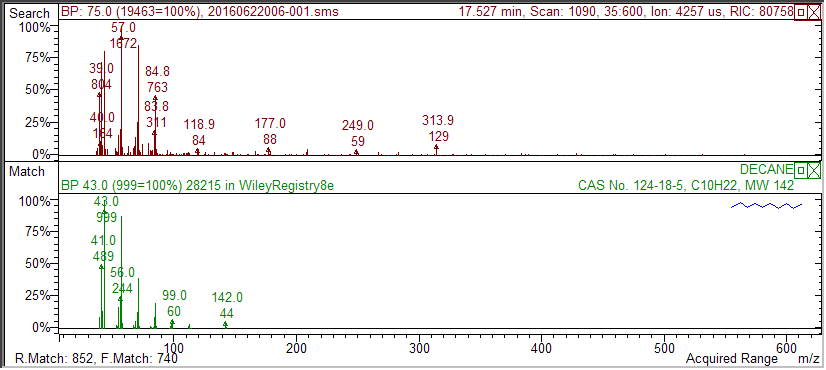
**

19. Glyceric acid


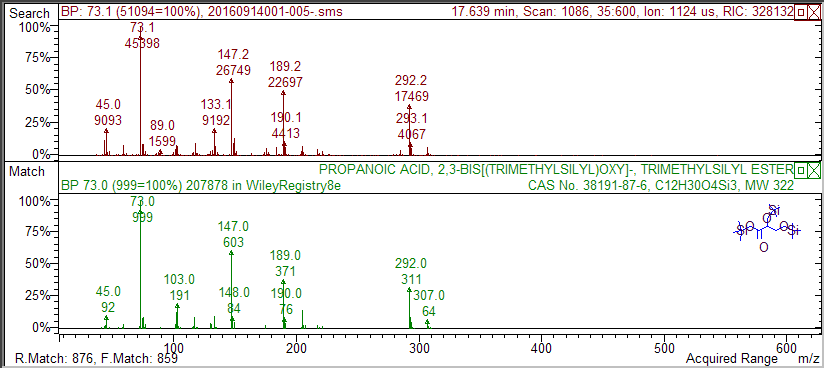


20. Fumaric acid

**
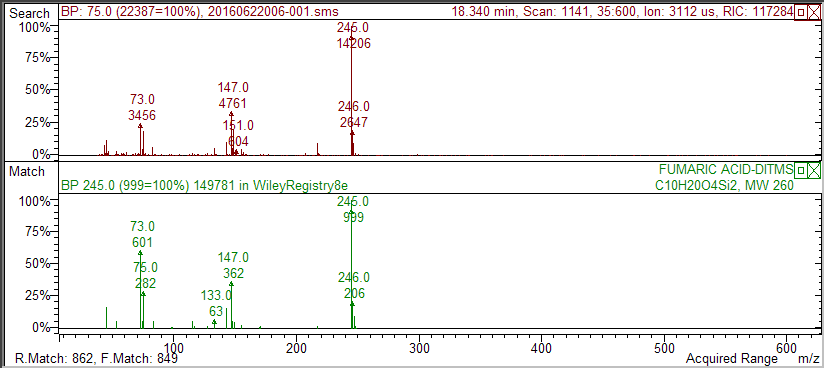
**

21. D-Erythronolactone

**
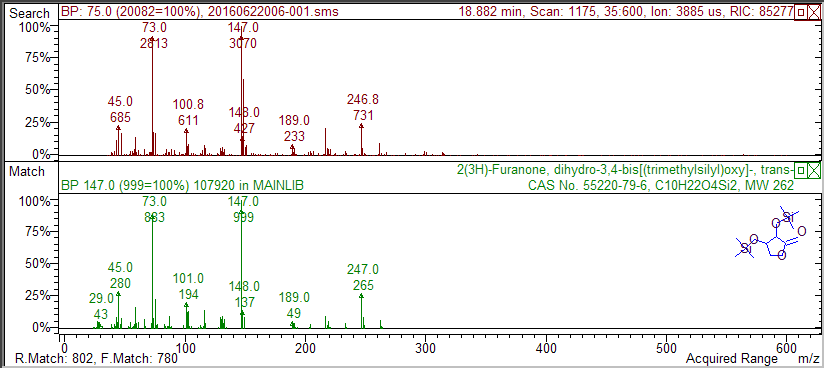
**

22. Threonine


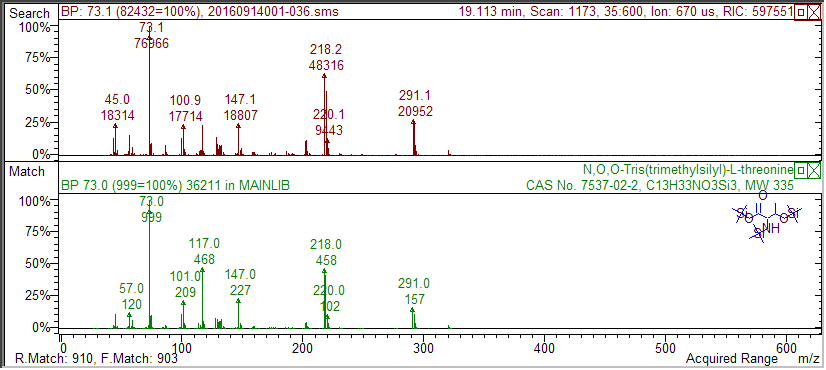


23. Malic acid


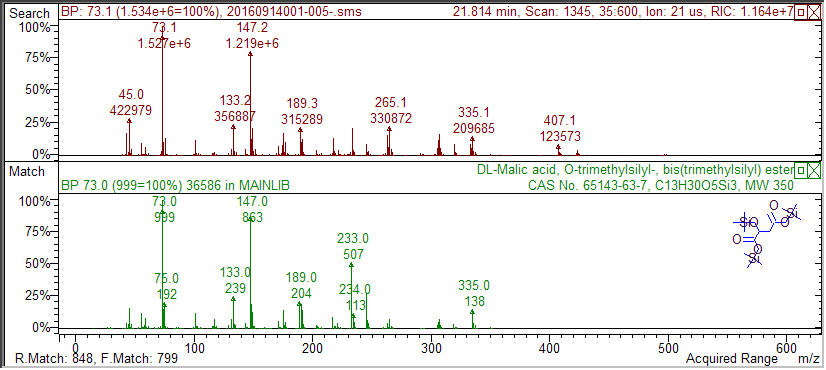


24. Dopamine


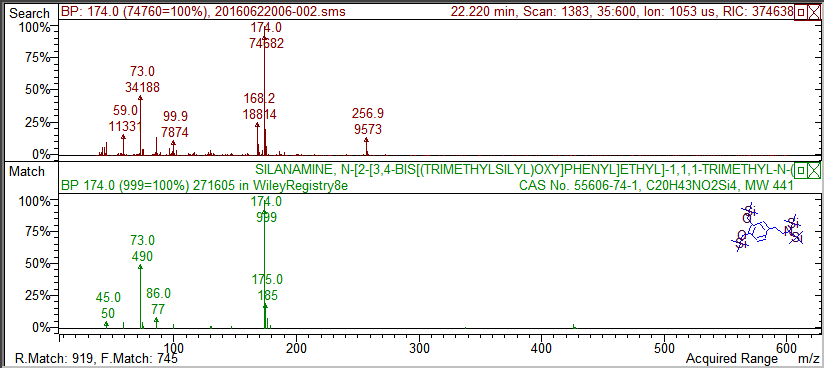


25. Aspartic acid

**
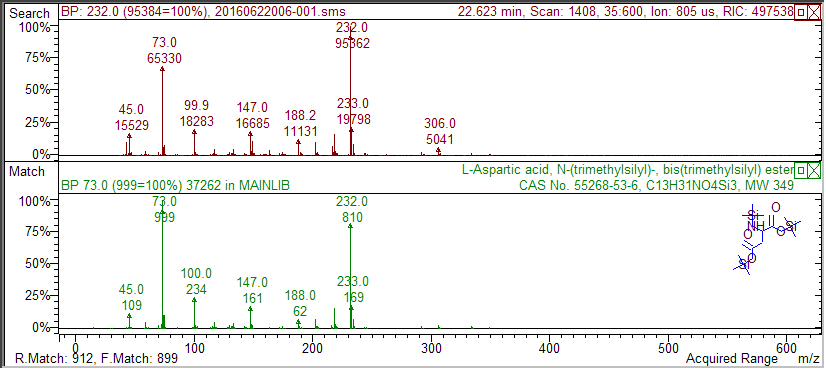
**

26. L-Pyroglutamic acid

**
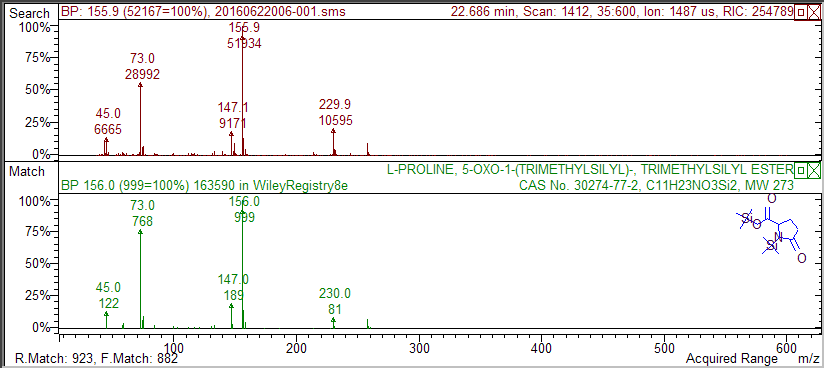
**

27. 4-Aminobutanoic acid

**
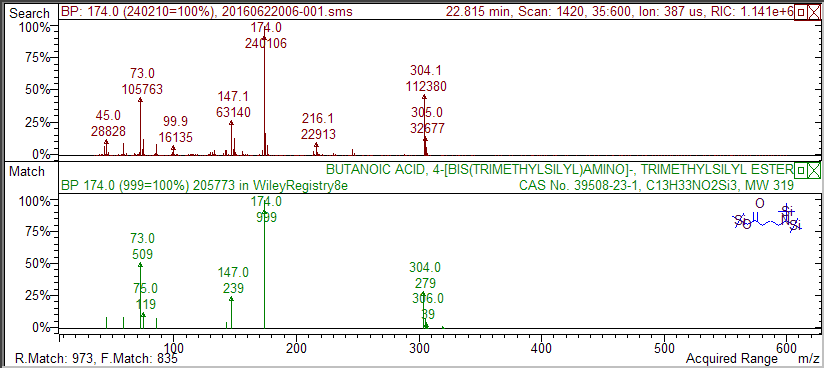
**

28. Threonic acid

**
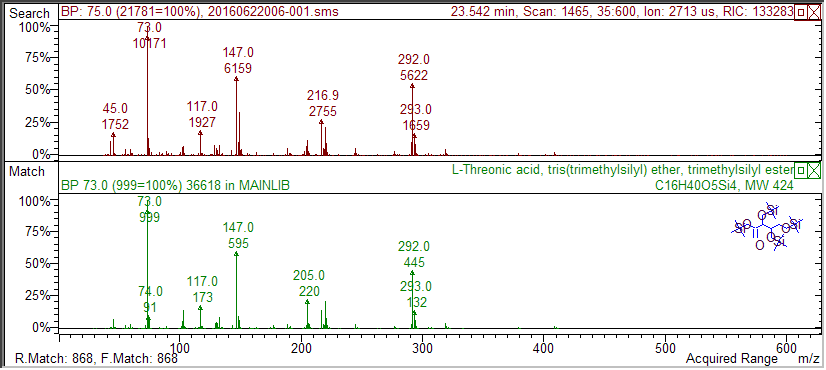
**

29. Erythritol

**
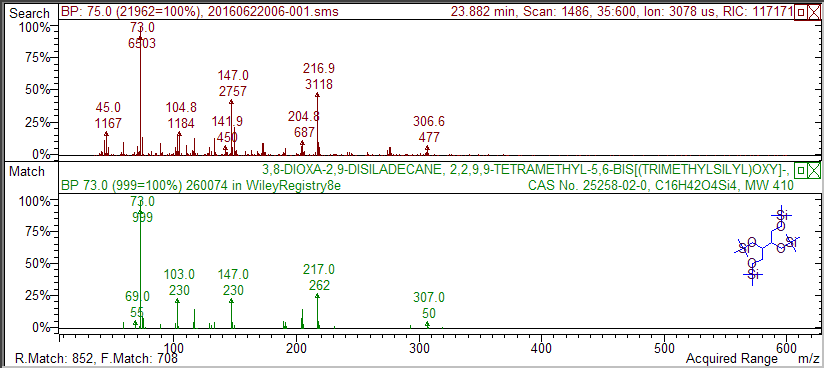
**

30. Arabinofuranose

**
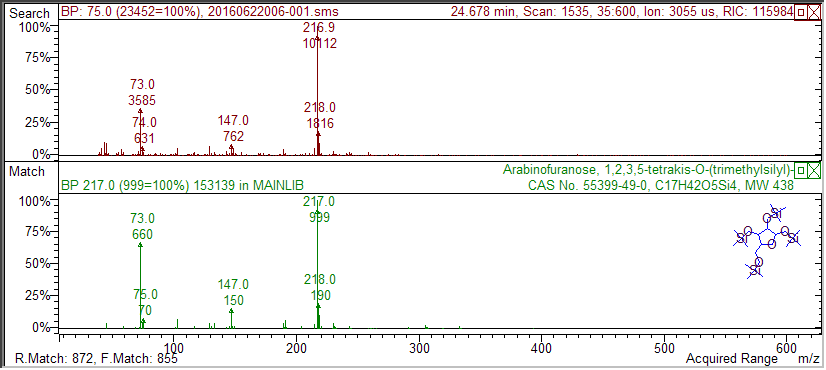
**

31. D-Xylono-1,4-lactone


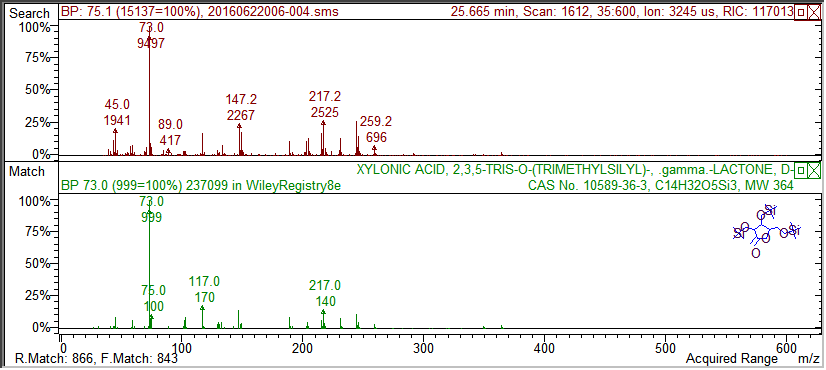


32. Nonadecane


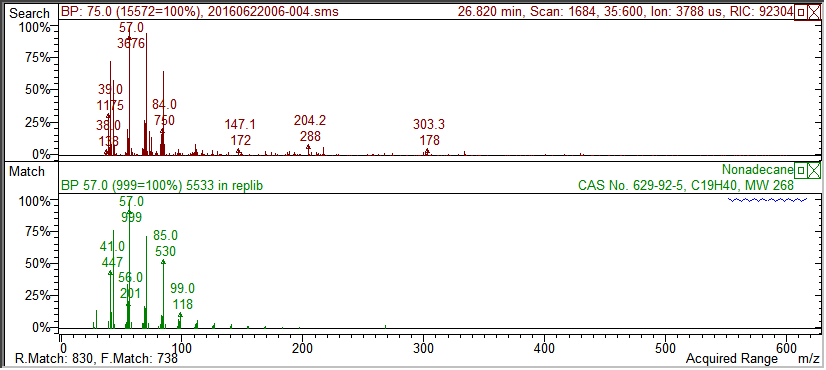


33. Putrescine

**
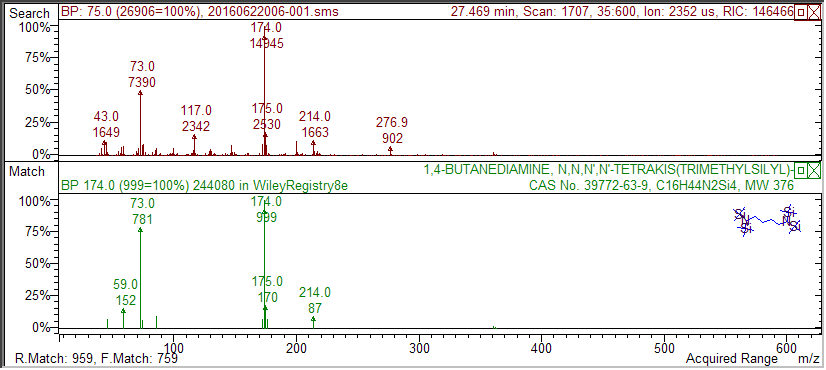
**

34. Glycerophosphoric acid

**
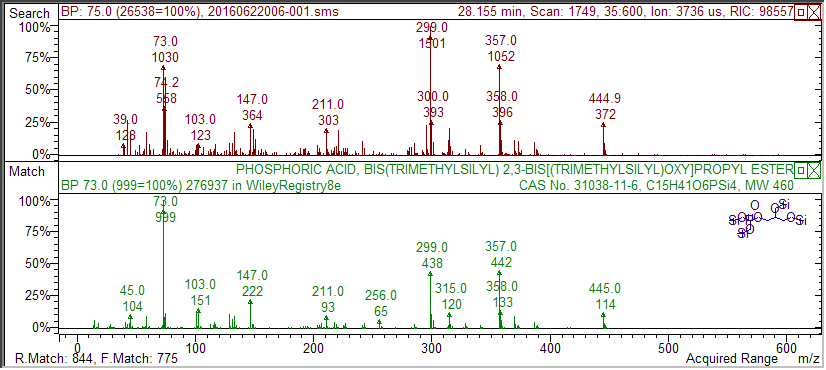
**

35. Azelaic acid

**
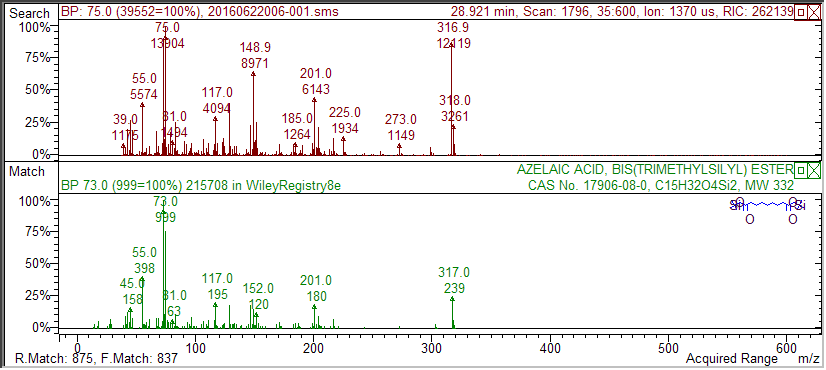
**

36. Citric acid

**
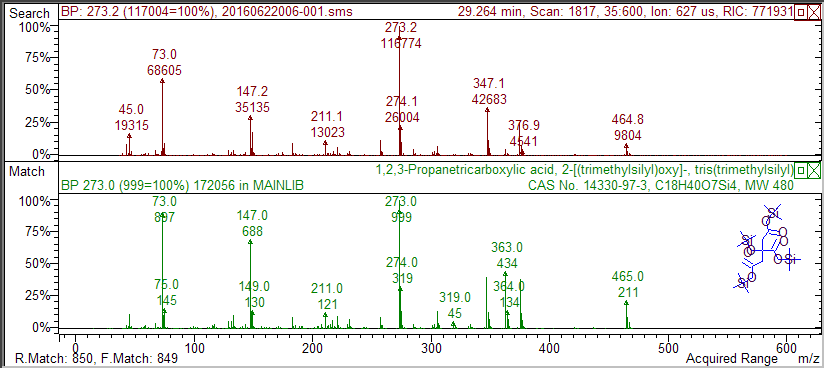
**

37. D-Xylofuranose

**
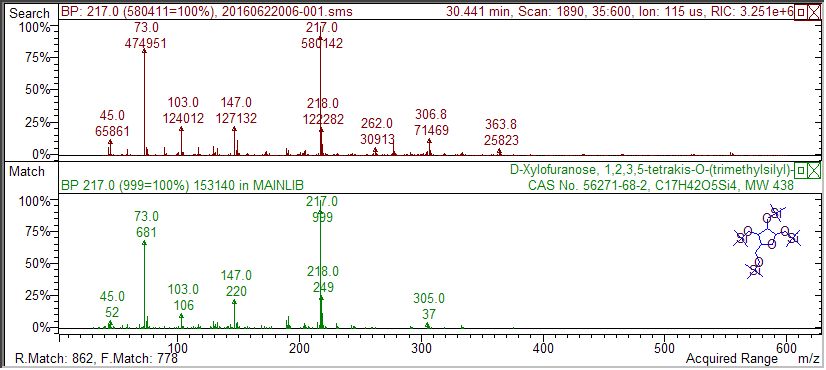
**

38. Syringic acid

**
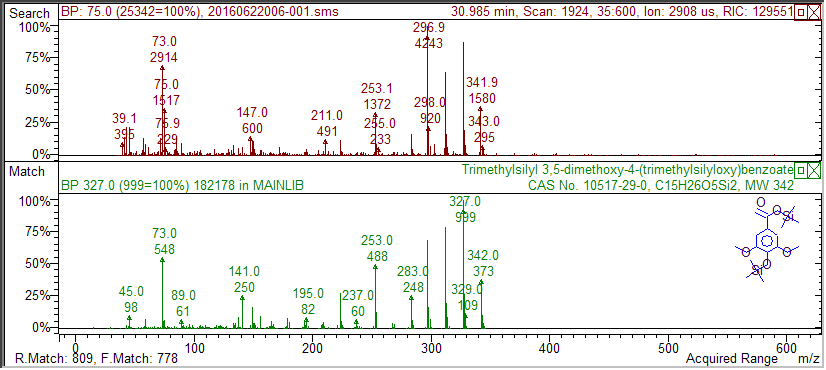
**

39. 7,9-Di-tert-butyl-1-oxaspiro(4,5)deca-6,9-diene-2,8-dione

**
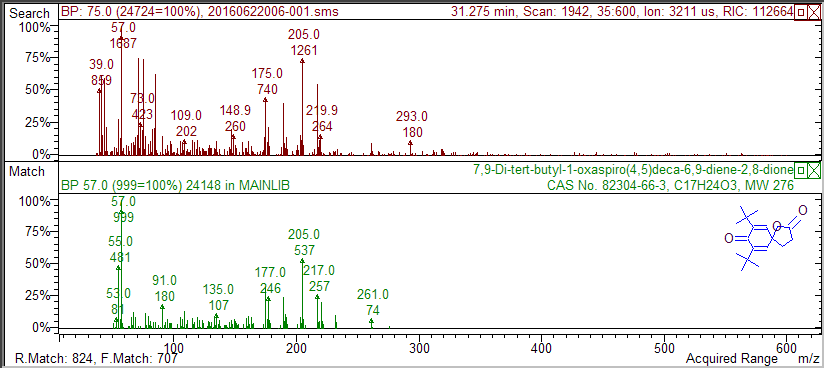
**

40. Glucaric acid

**
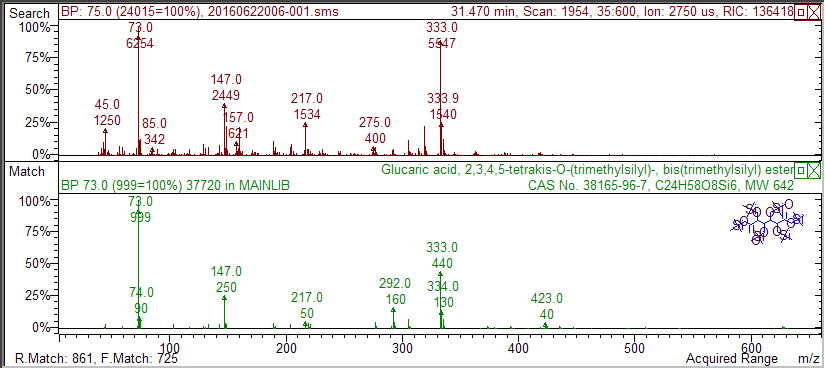
**

41. Hexadecanoic acid

**
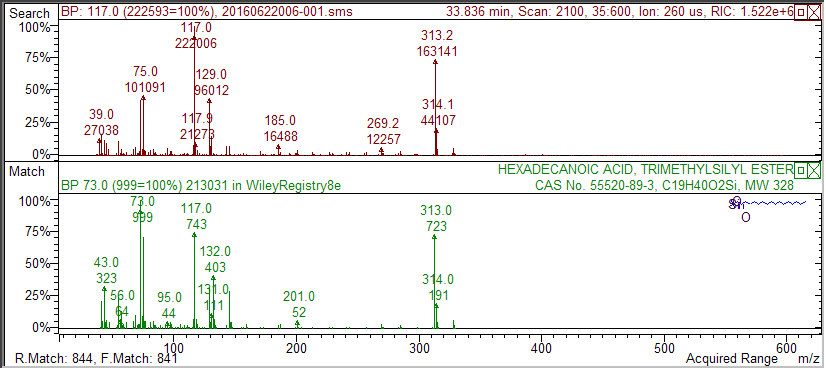
**

42. Stearic acid

**
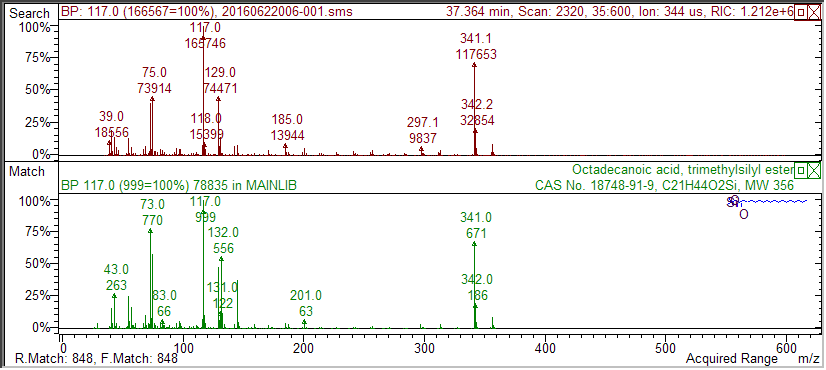
**

43. Arbutin

**
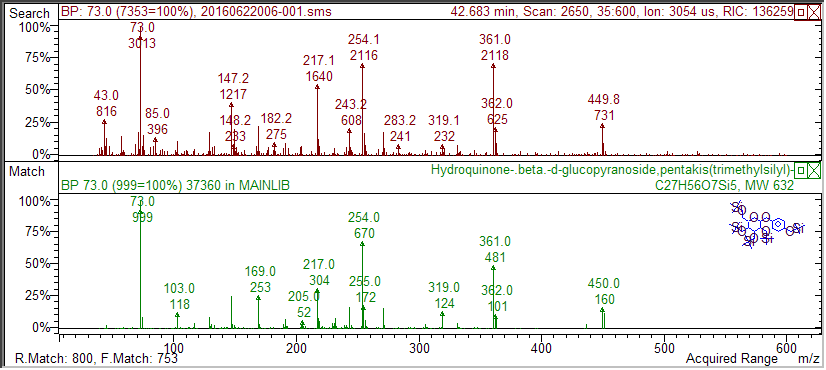
**

44. Sucrose

**
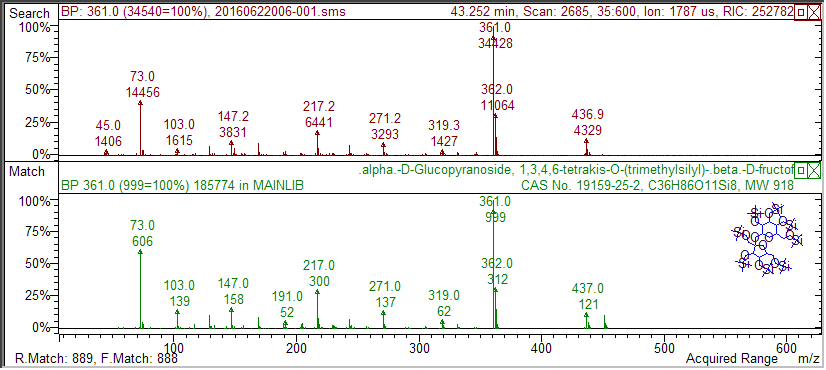
**
